# Development and characterization of functional sheep endometrial luminal epithelial organoids

**DOI:** 10.1186/s13567-026-01764-4

**Published:** 2026-06-09

**Authors:** Jiahe Guo, Xinai Huang, Rongxin Xia, Qin Gao, Hua Yang, Liqin Wang, Qingyang Mai, Mingtian Deng, Guomin Zhang, Yanli Zhang, Feng Wang

**Affiliations:** 1 Sanya Institute of Nanjing Agricultural University, Nanjing Agricultural University, Sanya, 572000 China; 2 Hu Sheep Academy, Nanjing Agricultural University, Nanjing, 210095 China; 3 Xinjiang Key Laboratory of Reproductive Regulation and Breeding for Ruminant Livestock, Urumqi, Xinjiang China; 4 College of Veterinary Medicine, Nanjing Agricultural University, Nanjing, China

**Keywords:** Luminal epithelial organoids, trophoblast proliferation, apical-out polarity, sheep endometrium, co-culture

## Abstract

The luminal epithelium (LE) serves as the primary interface for embryo–maternal communication during implantation. In ruminants such as sheep, the LE orchestrates critical reproductive events, including noninvasive adhesion, conceptus elongation, and pregnancy recognition. However, existing models face significant limitations: conventional two-dimensional (2D) cultures fail to recapitulate endometrial complexity, while current organoid systems are predominantly derived from glandular epithelium. Herein, we report the establishment of a robust three-dimensional (3D) culture system for generating ovine endometrial luminal epithelial organoids using an optimized expansion medium (ExM) supplemented with CHIR99021, Y-27632, SB202190, and the EphrinA1 ligand. The resulting organoids exhibited characteristic luminal epithelium features, including polarized architecture, mucin secretion, and high expression of lineage markers (keratin 18 (KRT-18), trophoblast cell surface antigen 2 (TROP2), and epithelial cell adhesion molecule (EpCAM)). Moreover, we developed an apical-out polarity model that faithfully recapitulates the in vivo luminal orientation. Transcriptomic analysis confirmed the close resemblance between organoids and native luminal epithelium, as well as maintained hormonal responsiveness. Functional validation demonstrated the organoids’ capacity to promote blastocyst expansion and trophoblast proliferation in co-culture systems, as well as substrate-specific adhesion that was enhanced by β-estradiol (E2) + medroxyprogesterone acetate (MPA) treatment. Notably, we identified erythropoietin-producing hepatocellular receptor A (EphA) signaling as a novel regulator of stemness properties during organoid development. The successful generation of functional luminal epithelial organoids thus provides a physiologically relevant model that mirrors native endometrial structure, hormone responses, and embryo interactions. As such, it offers a valuable platform for investigating the mechanisms of endometrial receptivity and for screening potential therapeutics aimed at improving reproductive efficiency in ruminants.

**Supplementary Information:**

The online version contains supplementary material available at 10.1186/s13567-026-01764-4.

## Introduction

The success of embryo implantation is contingent upon the development of a receptive uterine state within a specific spatiotemporal window [1, 2]. The uterine luminal epithelium (LE) serves as the primary cellular gatekeeper, representing the first maternal interface physically encountered by the embryo within the uterine environment [3, 4]. As a critical component of the maternal interface, LE cells help create the specialized implantation microenvironment through their regulated expression of adhesion molecules, secretion of chemokines, and release of exosomes [5–8]. Consequently, the structural integrity, polarity status, and molecular signature of LE cells serve as key mediators of endometrial receptivity.

The sheep has emerged as an indispensable model for studying ruminant reproductive physiology, owing to its unique reproductive features such as conceptus elongation and interferon-tau-mediated maternal recognition of pregnancy [9–11]. Unlike the invasive implantation observed in humans and rodents, sheep embryos attach via a noninvasive mode of adhesion, relying critically on communication between the LE and trophoblast cells at specialized uterine structures known as caruncles [12]. Within this context, transient adhesion between the trophectoderm and the LE is recognized as the initiating step for subsequent trophoblast migration, which is essential for conceptus growth and elongation. Moreover, dynamic changes in LE gene expression between days 10 and 12 of pregnancy are considered a prerequisite for the rapid trophoblast proliferation that drives post-hatching elongation [13, 14]. A critical morphological transition follows by gestational day 16, characterized by the adhesion and fusion of trophoblast cells with the maternal luminal epithelium to form binucleate cells (BNCs)—a definitive milestone in placental development [15].

Investigating the structural and functional dynamics of LE cells during receptivity establishment can provide crucial insights into the regulatory mechanisms governing maternal–fetal interactions in ruminants, thereby advancing reproductive efficiency. Conventional two-dimensional (2D) monolayer cultures fail to recapitulate the three-dimensional (3D) physiological microenvironment of the endometrium, and the absence of basement membrane-derived mechanical stimuli and 3D extracellular matrix (ECM) biochemical gradients disrupts authentic cell–matrix interactions [16, 17]. Furthermore, primary uterine epithelial cells in 2D culture systems are prone to phenotypic drift, morphological alterations, and accelerated senescence/apoptosis, making long-term stable passaging difficult to achieve [18]. Compounding these issues, the lack of in vivo hormonal regulatory networks significantly impairs the cells’ dynamic responsiveness to estrogen/progesterone fluctuations. Consequently, such in vitro models cannot accurately simulate physiological cellular behaviors, particularly limiting investigations into spatiotemporal-specific signaling crosstalk at the embryo–maternal interface [19].

Organoids, serving as sophisticated three-dimensional (3D) in vitro models, are typically generated through either adult stem cell self-organization or directed differentiation of pluripotent stem cells. These systems have become invaluable tools for physiological and pathological studies across various tissues, with their primary advantage lying in the high-fidelity recapitulation of functional units of organs [20, 21]. In endometrial research, the successful implementation of organoid technology in species such as humans, mice, and pigs has led to a key consensus identifying a conserved set of factors—including Wnt agonists (WNT3A, R-spondin-1), epidermal growth factor (EGF), fibroblast growth factor 10 (FGF10), and Noggin—as essential for the formation and maintenance of these cultures [22–25]. However, the field remains dominated by glandular epithelium-derived models, often characterized by a majority of FOXA2-positive cells. This predominance has resulted in the comparative underdevelopment of luminal epithelium-specific organoids, highlighting a critical gap [26]. This gap is primarily attributable to several technical hurdles: First, the inherent scarcity of luminal epithelial cells in the endometrium, as evidenced by single-cell RNA sequencing data showing their lower proportion and total count compared with glandular epithelial cells, presents a fundamental isolation challenge that results in organoid cultures predominantly composed of glandular epithelium [27, 28]. Second, luminal epithelium demonstrates significantly reduced stem cell potential compared with its glandular counterpart. This is evidenced by the markedly lower proportion of LGR5-positive cells in luminal populations, which directly correlates with their diminished expansion capacity in vitro [29]. Third, conventional protease digestion protocols lack the specificity to effectively discriminate between these two epithelial subtypes. Consequently, most current endometrial organoid systems inevitably produce mixed-cell populations containing both luminal and glandular derivatives [26, 30].

To date, no studies have reported the successful development of luminal epithelial organoids in ruminants. Notably, the caruncular regions of ruminant endometria possess a distinctive histological advantage—their natural absence of glandular structures provides an unparalleled source for isolating pure luminal epithelial populations [15]. This unique anatomical characteristic effectively circumvents the common contamination by glandular epithelium that plagues organoid derivation in other species.

This study established functional luminal epithelial organoids from ovine uterine caruncles. We demonstrate that these organoids recapitulate hallmark features of native LE, including the expression of definitive markers (KRT18, EpCAM, and TROP2), apical-basal polarity, and hormonal responsiveness mediated by steroid receptors. Crucially, functional assays confirmed their capacity to support blastocyst development and trophoblast proliferation. This novel model provides a powerful, physiologically relevant in vitro platform to explore the cellular determinants and regulatory mechanisms underlying endometrial receptivity in ruminants.

## Materials and methods

### Ethical statement

All animal procedures were carried out under strict adherence to the Basel Declaration, and the study protocol was approved by the Ethics Committee of Nanjing Agricultural University, China (protocol no. SYXK2022-0031). All experiments in this study were designed and analyzed on the basis of biological replicates. Biological replicates are defined as samples derived from different individual ewes. Unless specified as technical replicates, all reported *n* values refer to the number of independent biological replicates.

### Uterine tissue collection

Uterine tissues were collected from three primiparous Chinese Hu ewes (ages 396, 413, and 427 days; mean ± SD: 412 ± 15.52 days) at estrus phase (determined by ram testing) at a local abattoir. All three ewes had one previous pregnancy history with twin births. Within 2 h post-slaughter, tissues were transported on ice to the laboratory. Under sterile conditions, tissues were rinsed thoroughly with ice-cold phosphate-buffered saline (PBS) (C0221A, Beyotime) and longitudinally incised to expose the endometrial surface. Apical caruncular tissues (~5 mm × 5 mm) were meticulously dissected from each uterine horn (four explants per horn), avoiding intercaruncular regions to ensure luminal epithelial purity. The explants were immediately processed for enzymatic digestion. To serve as morphological and immunofluorescence controls, histologically intact endometrial tissues (containing a mixture of glandular, luminal, and stromal compartments) from regions adjacent to the dissected caruncles were collected and fixed in 4% paraformaldehyde (P0099, Beyotime). This standardized procedure ensures consistent isolation of high-purity luminal epithelium while preserving tissue integrity for downstream analyses. All organoid lines were derived from these three biologically independent individual sheep.

### Isolation, culture, and analysis of ovine endometrial luminal epithelial organoids

Organoid lines were established from endometrial tissues of three biologically independent adult Chinese Hu ewes, with each animal representing a distinct biological replicate and source of an independent organoid culture line. All subsequent experiments, including organoid formation efficiency analysis, immunofluorescence staining, and RNA sequencing (RNA-seq), were conducted using organoid lines derived from these three individual animals.

The enzymatic dissociation of ovine endometrial caruncles was adapted from established protocols [25, 31]. Briefly, excised caruncular tissues were minced into ~0.5 mm^3^ fragments and digested in Roswell Park Memorial Institute (RPMI) 1640 medium (12633012, Thermo Fisher Scientific) containing 2 mg/mL collagenase I (SCR103, Sigma), 0.5 mg/mL DNase I (260913, Sigma), and 0.1 mg/mL hyaluronidase (H3506, Sigma) at 37 °C for 60 min with gentle agitation. The resulting cell suspension was filtered through a 100-μm cell strainer (31752, Corning) and washed with Hank’s balanced salt solution (HBSS) buffer (H1025, Solarbio). For cryopreservation, organoids were harvested, resuspended in specialized freezing medium (HY-K6014, MCE), and stored at −80 °C.

The basal culture medium was adapted from the established, chemically defined formulation for human endometrial organoids [25]. Its composition was as follows: Advanced Dulbecco’s modified Eagle medium (DMEM)/F12 supplemented with 1 × N2, 1 × B27 (minus vitamin A), 100 μg/mL penicillin–streptomycin, 1.25 mM *N*-acetyl-L-cysteine, 50 ng/mL recombinant human EGF, 100 ng/mL recombinant human Noggin, 200 ng/mL recombinant human R-spondin-1, 100 ng/mL recombinant human FGF10, 50 ng/mL recombinant human HGF, 500 nM A83-01, and 10 mM nicotinamide. To identify additional key factors supporting organoid growth, we performed a systematic screen. Nine test formulations (T1–T9) were generated by supplementing this basal medium with different combinations of four pathway modulators: the WNT activator CHIR99021 (2 µM), the Rho-associated protein kinase (ROCK) inhibitor Y-27632 (10 µM), the p38 mitogen-activated protein kinase(MAPK) inhibitor SB202190 (1 µM), and the recombinant ligand EphrinA1-Fc (200 ng/mL). The presence ( +) or absence (−) of each component in the nine formulations is detailed in Table 1, a design that allowed assessment of individual and synergistic contributions. On the basis of the aforementioned screening, the combination of all four modulators was selected to formulate the final expansion medium (ExM) for routine culture. Therefore, ExM consists of the basal medium supplemented with 10 µM Y-27632, 32 µM CHIR99021, 1 µM SB202190, and 200 ng/mL preclustered recombinant human EphrinA1-Fc (see Table 2 for complete composition).

**Table 1:** **Formulations used in the organoid culture condition screening (T1–T9)**

Formulation	WNT3A (200 ng/mL)	CHIR99021 (2 µM)	Y-27632 (10 µM)	SB202190 (1 µM)	EphA1 (100 ng/mL)
T1	+	−	−	−	−
T2	−	+	−	−	−
T3	+	+	−	−	−
T4	+	+	+	−	−
T5	−	−	+	+	+
T6	+	+	−	+	−
T7	+	+	+	+	−
T8	+	+	+	+	+
T9	+	+	−	−	+

All formulations are based on the basal medium. “+” indicates inclusion of the component at the specified concentration

**Table 2:** **Details of organoid expansion medium (ExM)**

Product	Company	Product number	Final concentration
Advanced DMEM/F12	GIBCO	12634010	1 ×
N2 supplement	GIBCO	17502048	1 ×
B27 supplement minus vitamin A	GIBCO	12587010	1 ×
Penicillin and streptomycin	GIBCO	15140148	100 μg/mL
*N*-acetyl-l-cysteine	MCE	HY-B0215	1.25 mM
Recombinant human EGF	SinoBiological	10605-H01H	50 ng/mL
Recombinant human Noggin	SinoBiological	10267-HNAH	100 ng/mL
Recombinant human Rspondin-1	SinoBiological	11083-H08H	200 ng/mL
Recombinant human FGF10	SinoBiological	10573-HNAE	100 ng/mL
Recombinant human HGF	SinoBiological	10463-HNAS	50 ng/mL
A83-01	MCE	HY-10432	500 nM
Nicotinamide	MCE	HY-B0150	10 mM
WNT3A	SinoBiological	WNT007-02H	200 ng/mL
CHIR99021	MCE	HY-10182	2 μM
Y-27632	MCE	HY-10071	10 μM
SB202190	MCE	HY-10295	2 μM
Recombinant human Ephrin-A1	SinoBiological	10882-H02H	100 ng/mL

Organoid formation efficiency (OFE) was evaluated at passage 3. Briefly, 10 000 viable cells were seeded per Matrigel dome. On day 9, organoids were quantified by examining ten randomly selected, non-overlapping fields of view per dome at 10× magnification; only structures with a clear lumen and diameter ≥ 50 µm were counted as valid. The organoid count (or diameter) for each biological replicate (i.e., each Matrigel dome derived from one animal) was calculated as the mean value across these ten fields. This quantification was performed for each of the three biologically independent animals (*N* = 3 ewes). For cryopreservation, organoids were harvested, resuspended in specialized freezing medium, and stored at −80 °C.

### Generation of apical-out polarity in endometrial luminal epithelial organoids

To initiate polarity inversion, organoids were first liberated from Matrigel using Cell Recovery Solution in expansion medium. The organoid suspension was centrifuged at 200 × *g* for 3 min at room temperature and washed twice with Advanced DMEM/F12 (12634010,Gibco). For suspension culture, organoids were seeded into 24-well ultralow-attachment plates (3473,Corning) at a density of 50 organoids per well in 500 μL of organoid culture medium. To ensure uniform distribution, plates were gently swirled immediately after seeding. During the suspension culture period, organoid clusters were mechanically dispersed twice daily using wide-bore pipette tips to prevent excessive aggregation. Medium replacement was performed every 3 days through the following protocol: organoid suspensions were transferred to conical tubes using wide-orifice pipettes, allowed to settle by gravity for 5 min (or alternatively centrifuged at 200 × *g* for 30 s), followed by careful aspiration of 80% supernatant volume. The organoid pellets were then resuspended in fresh prewarmed medium and replated into new ultralow-attachment plates.

### Hormone treatment of endometrial luminal epithelial organoids

Ovine endometrial luminal epithelium organoids were treated with 10 nM β-estradiol (E2, Sigma E4389), 1 μM medroxyprogesterone acetate (MPA) (MCE HY-B0469), and 10 ng/mL recombinant Bovine Interferon τ (IFN-tau, HY-P71798, MCE) on the basis of established concentrations [32–34]. Following 4 days of basal culture (days 0–4) and polarity reversal on day 4, organoids were subjected to the following treatments from day 4 to 12: control (dimethyl sulfoxide (DMSO) vehicle, no hormones), E2 (10 nM from Day 6), E2 + MPA (E2 from day 6 plus 1 μM MPA from day 8), and E2 + MPA + IFN-tau (E2 and MPA as above, with 10 ng/mL IFN-tau added from day 10). All treatments contained ≤ 0.01% DMSO (D2650, Sigma) as vehicle. The medium was replaced every 24 h under standard culture conditions (37 °C, 5% CO_2_).

### HE and PAS staining

Endometrial organoids and tissue samples were fixed in 4% paraformaldehyde (tissues for 24 h, organoids for 30 min at 4 °C), followed by paraffin embedding. Organoids were pre-embedded in 1% agarose (Melford, MB1200) prior to paraffin embedding. Sections of 4 μm thickness were prepared for histological staining. The hematoxylin and eosin (H&E) staining procedure included deparaffinization and rehydration, hematoxylin staining for 5 min, differentiation and bluing, eosin staining for 1 min, dehydration and clearing, and mounting. The periodic acid–Schiff (PAS) staining procedure included oxidation with 0.5% periodic acid for 10 min, incubation with Schiff’s reagent in the dark for 15 min, counterstaining with hematoxylin, dehydration, and mounting. All stained sections were examined using a Nikon (ECLIPSE Ti2) optical microscope. Image analysis was performed using CASEVIEWER (version 2.4).

### Immunofluorescence

Basal-out and apical-out organoids were fixed at room temperature with 4% paraformaldehyde (P0099, Beyotime) for 30 min, followed by multiple washes with PBS (C0221A, Beyotime). The organoids were then permeabilized with 0.5% Triton X-100 (AGEL3585, Genie) in PBS for 20 min and washed thoroughly with PBS. Subsequently, samples were incubated in PBS containing 5% bovine serum albumin (BSA) at room temperature for 1 h to block nonspecific binding. After removing the blocking solution, primary antibodies diluted in 1% BSA/PBS (see Table 3 for details) were added and incubated overnight at 4 °C. Negative controls were performed under identical conditions by incubating samples with 1% BSA/PBS without primary antibodies (Additional file 4).

**Table 3:** **Details of antibodies used**

Antibodies	Cat. no.	RRID	Source	Dilution of IF	Dilution of western blot	Host species	Clonality
PGR	A0321	AB_2757125	ABclonal, Wuhan, China	–	1:1000	Human, mouse, rat	Polyclonal antibody
SPP1	A5814	AB_2766566	ABclonal, Wuhan, China	–	1:1000	Human, mouse, rat	Polyclonal antibody
TGFB1	A23262	AB_2761987	ABclonal, Wuhan, China	–	1:1000	Mouse, rat	Polyclonal antibody
MMP-2	A1144	AB_2766854	ABclonal, Wuhan, China	–	1:1000	Human, mouse, rat	Polyclonal antibody
PCNA	A13336	AB_2760192	ABclonal, Wuhan, China	–	1:1000	Human, mouse, rat	Polyclonal antibody
CCND1	A1301	AB_2759856	ABclonal, Wuhan, China	–	1:1000	Human, mouse, rat	Polyclonal antibody
CDK4	A23521PM	AB_2757151	ABclonal, Wuhan, China	–	1:1000	Human, mouse, rat	Polyclonal antibody
KRT-18	A1022	AB_2744510	ABclonal, Wuhan, China	1:200		Human, mouse, rat	Polyclonal antibody
EpCAM	A1177	AB_2758746	ABclonal, Wuhan, China	1:200		Human, mouse, rat	Polyclonal antibody
TROP2	A8129	AB_2772506	ABclonal, Wuhan, China	1:200		Human, mouse, rat	Polyclonal antibody
SOX9	67439-1-Ig	AB_2882675	Proteintech, Wuhan, China	1:200		Human, mouse, rat, pig, rabbit, canine	Monoclonal antibody
FOXJ1	86070-1-RR		Proteintech, Wuhan, China	1:400		Human	Monoclonal antibody
KRT-7	A4357	AB_2863248	ABclonal, Wuhan, China	1:200		Human, mouse, rat	Monoclonal antibody
GATA3	ab182747	AB_2924543	Abcam, Shanghai, China	1:200		Rat	Polyclonal antibody
FABP3	A5312	AB_2766124	ABclonal, Wuhan, China	1:200		Human, mouse	Polyclonal antibody
ZO-1	ab307799	AB_2924544	Abcam, Shanghai, China	1:100		Human, mouse, dog, cat, rat	Polyclonal antibody
β-Actin	AC026	AB_2768234	ABclonal, Wuhan, China	–	1:5000	Human, mouse, rat, chicken, zebrafish, pig, cow	Monoclonal antibody

Following several PBS washes, secondary antibodies provided by Thermo Fisher Scientific were applied at a dilution of 1:400, including Alexa Fluor 488 goat anti-mouse IgG1 (A21121), Alexa Fluor 568 goat anti-rabbit (A11011), or Alexa Fluor 647 (A21244). Nuclei were counterstained with 1 µg/mL 4′,6-diamidino-2-phenylindole (DAPI) (Sigma, D9542). After staining, samples were washed three times with PBS, 5 min each. The organoids were then transferred to confocal dishes and imaged using a Zeiss confocal microscope with ZEN software. Tissue sections were processed using the same staining protocol. Image analysis was performed using CASEVIEWER (version 2.4).

### Electron microscopy

For ultrastructural analysis, the fixation protocol was optimized to ensure optimal preservation of organoid morphology and ultrastructure. The following protocol was ultimately selected and used for all samples presented in this study: Organoids were fixed in 0.5% glutaraldehyde in 0.2 M sodium cacodylate buffer (pH 7.2) for 30 min, followed by post-fixation in reduced osmium tetroxide (1% OsO_4_ with 1.5% potassium ferrocyanide) for 60 min at room temperature, and then in 0.5% magnesium uranyl acetate for 16 h at 4 °C. After fixation, all samples were dehydrated through a graded ethanol series (70–100%), transitioned with acetonitrile (2×), and embedded in Quetol epoxy or Epon resin. Ultrathin sections (70 nm) were prepared using a Leica UCT ultramicrotome. Transmission electron microscopy (TEM) was performed on a JEOL JEM-1400 microscope, and images were acquired with an Olympus SIS Quemesa 11-megapixel digital camera system.

### Western blot analysis

Western blot analysis was performed according to our previously described procedure, with minor modifications [35]. Briefly, proteins were extracted from tissues and cells using radioimmunoprecipitation assay (RIPA) buffer and quantified with a bicinchoninic acid (BCA) Protein Assay Kit (P0012S, Beyotime). A total of 20 µg of protein was loaded onto a 12% sodium dodecyl sulfate (SDS)-polyacrylamide gel electrophoresis (PAGE) gel (AP1002-50, Applygen) and transferred to polyvinylidene fluoride (PVDF) membranes (PB9220, Invitrogen). After blocking with 5% nonfat milk, the membranes were incubated overnight at 4 °C with primary antibodies (Table 3), followed by incubation with a secondary antibody for 1 h. Protein signals were detected using an enhanced chemiluminescence kit (K10001, Proteintech) and visualized with a detection system (Fujifilm, Tokyo, Japan), and the images were analyzed using ImageJ software.

### RNA isolation and quantitative real-time polymerase chain reaction (qRT-PCR) analysis

Total RNA was isolated from uterine tissues and endometrial epithelial cells using TRIzol reagent (Invitrogen). Genomic DNA was removed, and first-strand complementary DNA (cDNA) was synthesized from 1 µg of total RNA using the TransGen Biotech AU341 kit, according to the manufacturer’s instructions. The absence of genomic DNA (gDNA) contamination was confirmed by no-reverse transcription (no-RT) controls (Additional file 9). All primer sequences (Table 4) were validated for specificity and efficiency (90–110%, *R*^2^ > 0.98), and *ACTB* was selected for normalization based on RefFinder analysis. qPCR was performed using ChamQ Universal SYBR qPCR Master Mix (Vazyme, cat. no. Q711-02/03) on a QuantStudio™ 5 Real-Time PCR System (Thermo Fisher Scientific). The 20 µL reaction system consisted of 10 µL of 2 × ChamQ Universal SYBR qPCR Master Mix, 0.4 µL of each forward and reverse primer (10 µM), 1 µL of cDNA template, and was made up to 20 µL with nuclease-free water. All reactions, including no-template controls, were run in triplicate for each biological sample. The thermal cycling protocol consisted of an initial denaturation at 95 °C for 30 s, followed by 40 cycles of 95 °C for 10 s and 60 °C for 30 s. A melt curve analysis was performed at the end of each run to confirm the specificity of the amplified product. The average cycle threshold (Ct) value from the technical triplicates was used for analysis, and relative gene expression was quantified using the 2^–ΔΔCt^ method. All experiments were conducted with at least three independent biological replicates.

**Table 4:** **Primer sequences used for this study**

Item	Primer sequence (5′–3′)		Fragment size (bp)	Gene Bank no.
	Forward	Reverse		
PGR	CCTGTGGAAGCTGTAAGGTC	AGTTCCGAAAACCTCCAAGA	171	XM_027979146.3
SPP1	TCACAGGGGACTGGACTCTT	GGTTTAACTGGAAGGGCGGA	117	NM_001009224.1
TGFB1	ACACACAGTACAGCAAGGTCC	CACGTAGTACACGATGGGCA	113	NM_001009400.2
MUC1	TCTCATTGCCCTGGTTGTGT	TAGGGGCTCCGTTTGGTACT	156	XM_027976040.2
PCNA	TGCAGATGTACCCCTTGTTGT	CATCCTCGATCTTGGGAGCC	83	XM_004014340.5
CCND1	ATCAGATGTGACCCGGACTG	CCCTCAAATGTTCACGTCGC	183	XM_027959928.2
CDK4	CAGTGTACAAGGCCCGTGAT	GACGTCCATGAGCCTGACAA	176	NM_001127269.1
MMP-2	CGCTTCCAGGGCACATCTTA	CAGTGGACATGGCAGTCTCG	133	NM_001166180.1
ACTB	TCAGCAAGCAGGAGTACGAC	ACGAGGCCAATCTCATCTCG	137	NM_001009784.3

### RNA-seq and bioinformatics analysis

Total RNA was isolated using TRIzol reagent (Invitrogen) and assessed on an Agilent 2100 Bioanalyzer; all samples had RNA integrity number (RIN) values > 9.0, and representative electropherograms are shown in Additional file 5. Libraries were prepared from 3 μg of total RNA using the NEBNext Ultra II Directional RNA Library Prep Kit for Illumina (New England Biolabs), which includes standard Illumina adapter sequences, and sequenced on an Illumina NovaSeq 6000 platform in paired-end 150-bp mode, yielding an average of 53.9 million raw reads per sample (range 43.4–59.5 million). Raw reads were processed with fastp version 0.22.0 to remove adapter sequences and low-quality reads (average quality < Q20); after filtering, the Q30 percentage was > 95% for all samples. Clean reads were aligned to the sheep reference genome ARS-UI_Ramb_v2.0 (GCA_016772045.1) using HISAT2 v2.1.0 with default parameters, on the basis of gene annotation from the National Center for Biotechnology Information (NCBI) RefSeq database. Gene expression was quantified with HTSeq v0.9.1, and raw read counts were processed for differential expression analysis in DESeq2 (|log_2_FC|≥ 1, adjusted *p* < 0.05). Hierarchical clustering was performed using the pheatmap R package (version 1.0.12) on the basis of Euclidean distance and complete linkage, and functional enrichment analysis was conducted with topGO (Fisher’s exact test, adjusted *p* < 0.05). Raw and processed RNA-seq data have been deposited in the China National GeneBank Sequence Archive (CNSA) under accession no. CNP0008511, and raw read counts are provided in Additional file 1.

### Generation of a luminal epithelial reference from scRNA-seq data

To construct a faithful transcriptional reference for validating the organoid model, a pseudo-bulk luminal epithelial (LE) transcriptome was generated from single-cell RNA sequencing (scRNA-seq) data. Briefly, the scRNA-seq data were derived from unpublished datasets of endometrial tissues from Chinese Hu sheep at gestational days 9, 12, and 16 (China National GeneBank database, accession no. PRJCA056151). Data processing and cell annotation were performed using Seurat (version 4.3.0). Luminal epithelial cells were robustly identified by high expression of canonical markers (KRT18 and EpCAM) and the absence of stromal (vimentin (VIM)) or glandular (FOXA2) markers. The raw gene counts from all annotated luminal epithelial cells across all samples were aggregated to create a single, high-quality pseudo-bulk reference profile. This aggregated pseudo-bulk LE reference transcriptome is provided in Additional file 2 and served as the native LE reference for subsequent correlation analysis with the bulk RNA-seq data from organoids.

### Isolation and culture of trophoblast cells and endometrial stromal cells

Trophoblast cells were isolated from ovine placentomes (gestational days 45–60) as previously described, with minor modifications [36]. Briefly, after dissecting and washing cotyledons in ice-cold PBS, they were minced into 1-mm^3^ pieces and digested with 2 mg/mL collagenase type I at 37 °C for 30 min (with shaking every 10 min). The digestate was filtered through a 75-µm cell strainer and centrifuged, and the cell pellet was washed with PBS. Cells were resuspended in DMEM/F12 complete medium, counted, and plated at a density of 5 × 10^5^ cells/mL. Cultures were maintained at 37 °C with 5% CO₂. Upon reaching 80–90% confluence, trophoblast cells were purified by differential trypsinization and characterized via positive immunostaining for Cytokeratin 7 (CK7) and GATA binding protein 3 (GATA3) (Additional file 6).

Endometrial stromal cells (ESCs) were isolated from nonpregnant ewes using an explant culture technique followed by differential trypsinization. Briefly, uterine horns were opened longitudinally, and the endometrial tissue was scraped off, minced into fine fragments (≤ 1 mm^3^), and washed. These tissue explants were then cultured in DMEM/F-12 medium supplemented with 20% fetal bovine serum. After 48 h of culture, both endometrial epithelial cells (EECs) and ESCs migrated out of the explants and began to proliferate. Upon reaching approximately 90% confluence, the cells were washed and briefly treated with 0.25% trypsin for 2 min at 37 °C. This short digestion was sufficient to detach the ESCs, which were then collected in the cell pellet after centrifugation. In contrast, the EECs remained predominantly adherent to the culture dish. The pelleted ESCs were reseeded for expansion. Successful isolation of ESCs was confirmed by immunofluorescence analysis, demonstrating that the cells were positive for the stromal marker VIM but negative for the epithelial cytokeratin KRT18.

### Co-culture of organoids with hatched blastocysts and trophoblast cells

In vitro embryo production was performed according to established protocols with minor modifications [32, 33].

Oocyte collection and in vitro maturation (IVM): Ovine ovaries were obtained from a local abattoir and transported to the laboratory in sterile saline at 30–35 °C. Cumulus–oocyte complexes (COCs) were aspirated from antral follicles (2–6 mm in diameter) using an 18-gauge needle. Only COCs with a homogeneous cytoplasm and surrounded by at least three layers of compact cumulus cells were selected. Groups of up to 50 COCs were cultured in 500-μL droplets of IVM medium for 24 h at 38.5 °C under 5% CO_2_ in air. The IVM medium consisted of TCM-199 (11150-059, Gibco) supplemented with 10% (v/v) fetal bovine serum (FBS, 10270-106, Gibco), 10 µg/mL follicle-stimulating hormone (FSH, F2293, Sigma), 10 µg/mL luteinizing hormone (LH, L5269, Sigma), and 1 µg/mL β-estradiol (E4389, Sigma).

In vitro fertilization (IVF): After maturation, COCs were washed and transferred into 100-μL fertilization droplets. Fresh ram semen from a fertile donor was used. Spermatozoa were selected by a swim-up procedure in Sperm-TALP medium. The final sperm concentration was adjusted to 1 × 10^6^ spermatozoa/mL. Gametes were co-incubated for 18–24 h at 38.5 °C under 5% CO_2_.

In vitro culture (IVC): Presumptive zygotes were denuded by gentle pipetting to remove cumulus cells. They were then washed and cultured in groups of 20–25 in 50-μL droplets of BO-IVC one-step culture medium (71005, Hua Yue Hang Instrument Co., Ltd.) under mineral oil. The culture was carried out at 38.5 °C in a humidified tri-gas incubator set to 5% CO_2_ and 5% O_2_, with N_2_ making up the remainder to mimic physiological oxygen tension. The culture medium was half-refreshed every 48 h. Blastocysts developed by day 7 or 8 post-fertilization were used for subsequent co-culture experiments with organoids.

Upon zona pellucida hatching on D8, hatched blastocysts (with eight biological replicates) were transferred to the center of low-adhesion 24-well plates containing 500 μL endometrial luminal epithelium organoid medium, with 100 apical-out organoids generated as described in Sect. 2.5 seeded at the periphery for co-culture. Following co-culture, the size of the blastocysts was determined using ImageJ software.

### Cell cycle analysis by flow cytometry

For cell cycle analysis, organoids were first dissociated into single cells by digestion with 0.25% trypsin (Gibco, 25200056) in Advanced DMEM/F12 (Gibco, 12634010) at 37 °C for 5 min, and the reaction was terminated with DMEM/F12 medium (Gibco, 11330032) supplemented with 5% fetal bovine serum (FBS; Gibco, 10270106). The harvested single cells were fixed in 70% ethanol at 4 °C overnight. After fixation, cells were incubated with phosphate-buffered saline (PBS) containing 100 µg/mL RNase A (Thermo Fisher, EN0531) at 37 °C for 30 min, followed by staining with 50 µg/mL propidium iodide (PI; Sigma-Aldrich, P4170) in the dark at room temperature for 30 min. The DNA content was analyzed using a BD FACSCanto II flow cytometer (BD Biosciences, USA). Data from at least 10 000 events per sample were collected and processed with the BD FACSCanto System II software (version 2.4) to determine the percentage of cells in the G0/G1, S, and G2/M phases.

### Assessment of cell adhesion at two levels: organoid and single-cell

We assessed organoid adhesion on four substrates: confluent monolayers of trophoblast cells or endometrial stromal cells (ESCs), fibronectin-coated wells (10 µg/mL), and standard tissue culture-treated plates. Fifty organoids were added to each well and allowed to adhere. Nonadherent organoids were removed by gentle washing with prewarmed PBS, which included rocking the plate at 50 RPM for 3–5 rotations (~4–6 s) on an orbital shaker and subsequent careful aspiration of the liquid. Adherent organoids were then quantified by microscopic counting. To assess cell adhesion, organoids subjected to various hormone treatments were dissociated into single cells via trypsinization. The cells were then plated on fibronectin-coated culture plates. Following a 6-h adhesion period, the medium was refreshed, and cell adhesion was quantified using a Cell Counting Kit-8 (CCK-8) assay. Optical density (OD) at 450 nm was measured and converted to actual cell numbers on the basis of a standard curve generated from known cell counts.

### Statistical analysis

All quantitative data in this study were derived from three biologically independent organoid lines, each established from endometrial caruncles of a separate ewe (*N* = 3). For the organoid–embryo co-culture experiments, eight embryos were used per group (*N* = 8). Data are presented as median with interquartile range. Given the sample sizes, nonparametric statistical tests were employed for all comparisons. The Mann–Whitney *U* test was used for comparisons between two groups. For comparisons involving multiple groups (including all qPCR and Western blot data), the Kruskal–Wallis test with Dunn’s post hoc test was applied. *p* < 0.05 was considered statistically significant. Significance is denoted as *p* < 0.05, *p < 0.01, and **p < 0.001, or by different superscript letters (e.g., a, b). All analyses were performed using SPSS Statistics (version 28.0.1.1).

## Results

### Optimization of ovine endometrial luminal epithelial organoid culture: protocol development and medium refinement

**Figure 1:**
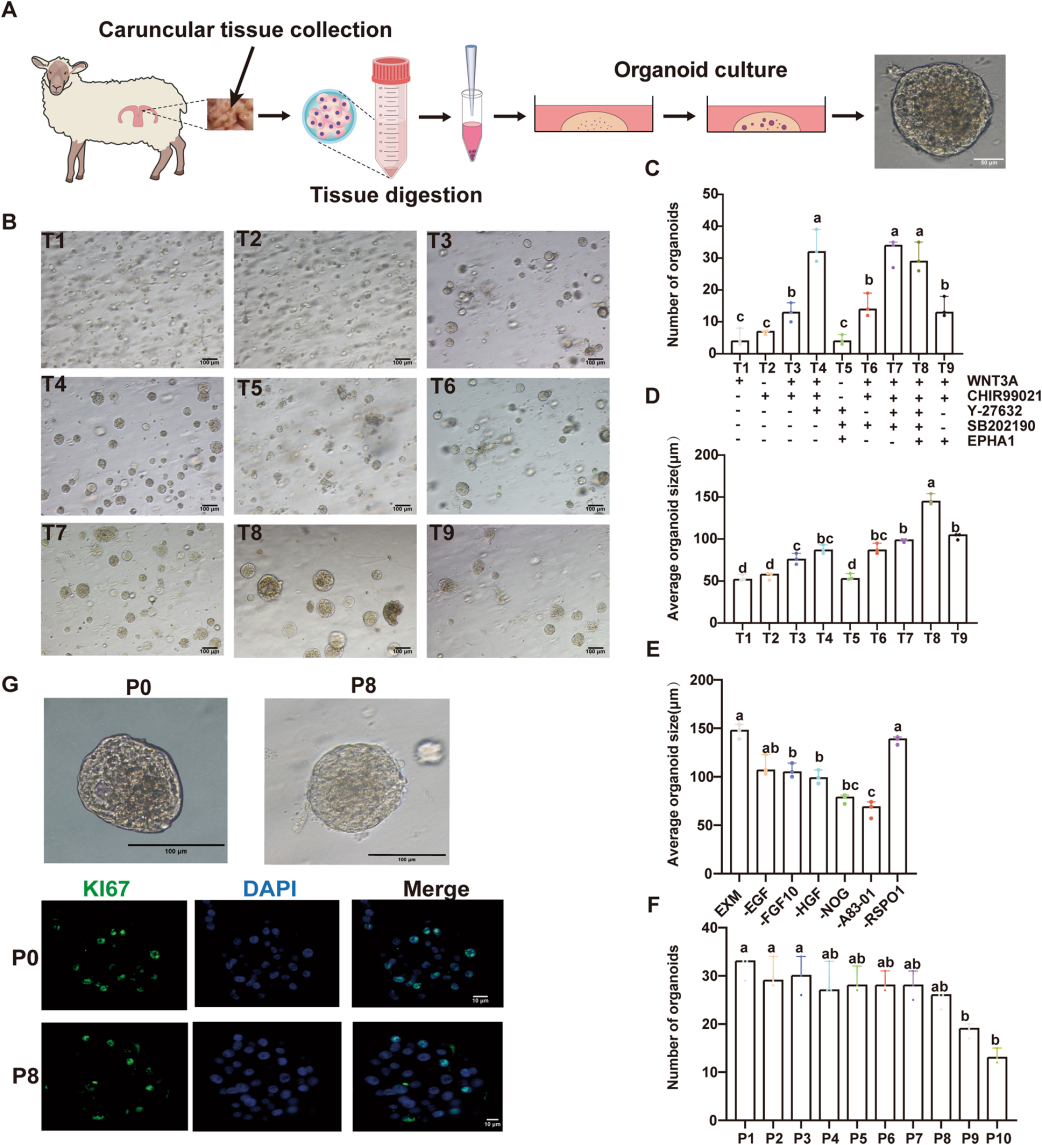
**Development and optimization of ovine endometrial luminal epithelial organoid culture system. A** Schematic diagram illustrating the isolation of luminal epithelial cells from ovine endometrial caruncles and subsequent 3D culture in Matrigel. **B** Screening of key signaling pathway activators. Organoids were cultured in basal medium (for composition, see “Methods”) supplemented with nine different formulations (T1–T9; for specific component combinations, see Table 2). Representative light microscopy images are shown. Scale bar: 100 µm. **C** Organoid yield under the conditions defined in Table 2 (T1–T9). **D** Average organoid diameter under the conditions defined in Table 2 (T1–T9). **E** Effect of individual growth factor withdrawal from the optimized expansion medium (ExM; see Table 1 for composition) on organoid diameter. **F** Quantitative analysis of organoid formation efficiency at different passage numbers (P0, primary culture; P8, passage 8). **G** Morphology of organoids at P0 and P8. Proliferation was assessed by Ki-67 immunofluorescence (IF) staining (green). Nuclei are counterstained with DAPI (blue). Scale bars: 100 µm (light microscopy), 10 µm (IF). For panels **C**–**F** Each data point represents one biological replicate (a Matrigel dome derived from one animal; *N* = 3 ewes). The value for each replicate was calculated as the mean from ten randomly sampled fields of view. Data are presented as median with interquartile range. Different superscript letters (a–e) denote statistically significant differences (*p* < 0.05, Kruskal–Wallis test with Dunn’s post hoc comparison). The schematic diagram in **A** was created with Microsoft PowerPoint (Office 2021).

We describe the development of a robust 3D culture system for generating ovine endometrial luminal epithelial organoids. The process began with the isolation of primary cells from endometrial caruncles, followed by their embedding in Matrigel for organoid development (Figure 1A). To define a suitable medium, we systematically screened key signaling pathway activators in a basal medium background. Nine different formulations (T1–T9) were evaluated, with representative morphological outcomes shown in Figure 1B. Quantitative analysis demonstrated that the combination of WNT pathway activation (using CHIR99021), together with ROCK inhibition (Y-27632), p38 MAPK inhibition (SB202190), and EphrinA1-Fc stimulation, yielded the highest organoid formation efficiency (Figure 1C) and significantly promoted organoid enlargement, achieving average diameters exceeding 100 μm (Figure 1D).

We further performed a growth factor withdrawal screen to identify essential components. Removal of EGF, NOG, FGF10, A83-01, or HGF from the expanded medium (ExM) markedly reduced organoid diameter, whereas omission of RSPO1 had no significant effect, confirming its dispensability in this system (Figure 1E). The optimized protocol enabled robust expansion, with organoids sustaining stable formation efficiency for at least eight passages before a marked reduction occurred (Figure 1F). Morphological examination combined with Ki-67 immunofluorescence confirmed that organoids retained structural integrity and high proliferative activity even at passage 8 (Figure 1G).

### Characterization of ovine endometrial luminal epithelial organoids

**Figure 2:**
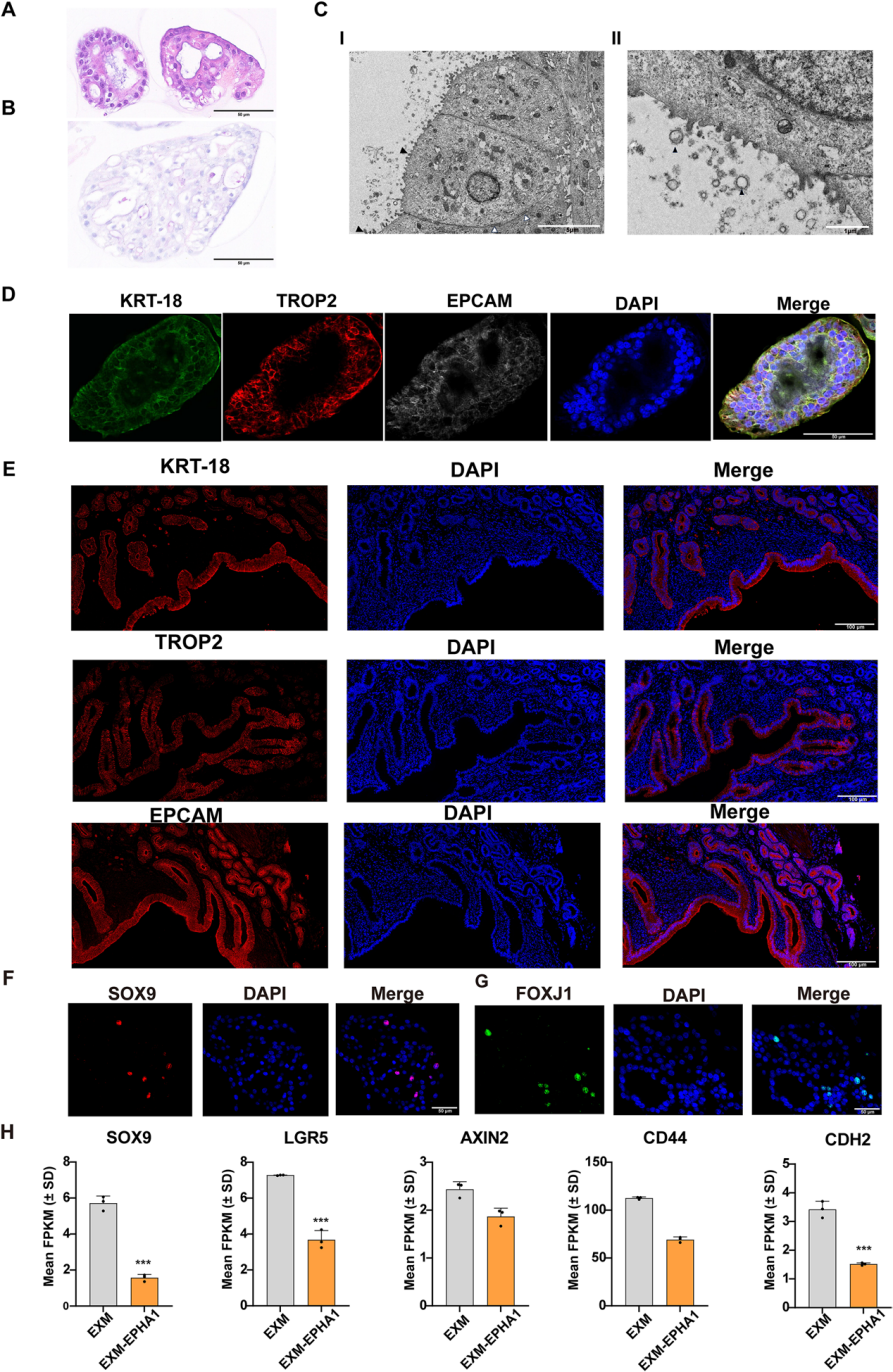
**Phenotypic and molecular characterization of endometrial organoids. A** Hematoxylin and eosin (H&E) and **B** periodic acid–Schiff (PAS) staining of endometrial luminal epithelial organoids. **C** Transmission electron microscopy (TEM) images revealing ultrastructural features: **CI** Apical microvilli (black arrows) and intercellular desmosomes (white arrows). Scale bar: 5 µm. **CII** Secretory vesicles (black arrows) localized at the base of microvilli. Scale bar: 1 µm. **D** Immunofluorescence (IF) staining confirms high expression of luminal epithelial markers: cytokeratin 18 (KRT-18), trophoblast cell surface antigen 2 (TROP2), and epithelial cell adhesion molecule (EpCAM). **E** Expression patterns of the same luminal epithelial markers (KRT-18, TROP2, and EpCAM) in native endometrial tissue. Scale bar: 100 µm. **F** Immunofluorescence (IF) staining for the transcription factor SRY-box transcription factor 9 (SOX9). **G** IF staining for the transcription factor forkhead box J1 (FOXJ1). For panels **A**, **B**, **D**, **F**, and **G** scale bars: 50 µm. **H** Expression of stem cell-related genes in organoids, with or without EphrinA1-Fc stimulation. Differentially expressed genes were identified by RNA sequencing (RNA-seq) analysis using DESeq2 with thresholds of adjusted *p*-value (false discovery rate, FDR) < 0.05 and absolute log_2_ fold change > 1. Data are presented as mean fragments per kilobase of transcript per million mapped reads (FPKM) ± standard deviation (SD) from three biological replicates (*N* = 3 independent organoid lines, each derived from one ewe). Significance levels: *FDR < 0.05, **FDR < 0.01, ***FDR < 0.001. The images shown in **A**–**G** are representative of three biologically independent experiments (organoid lines from three individual ewes).

Comprehensive phenotypic and molecular characterization was performed to validate the organoid model. Histological examination revealed that the organoids formed well-defined lumen-like structures, as shown by hematoxylin and eosin (H&E) staining (Figure 2A). Periodic acid–Schiff (PAS) staining confirmed functional maturation through abundant mucin production and secretion into the luminal space (Figure 2B). At the ultrastructural level, transmission electron microscopy (TEM) identified characteristic features of polarized epithelium, including dense apical microvilli, well-formed intercellular desmosomes, and numerous secretory vesicles (Figure 2C). Immunofluorescence analysis demonstrated strong expression of characteristic luminal epithelial markers including keratin 18 (KRT-18), trophoblast cell surface antigen 2 (TROP2), and epithelial cell adhesion molecule (EpCAM) (Figure 2D); critically, all organoids were negative for the glandular epithelial marker FOXA2 (Additional file 7). The expression patterns of these markers closely mirrored their spatial distribution in native endometrial tissue (Figure 2E), confirming the physiological relevance of the organoid model. Immunofluorescence confirmed the presence of forkhead box J1 (FOXJ1), a ciliated cell marker, indicating the existence of a ciliated cell lineage (Figure 2G). The expression of SRY-box transcription factor 9 (SOX9), a key stemness-associated transcription factor, was also detected, confirming the presence of a stem cell population (Figure 2F). Transcriptomic analysis revealed the expression of a panel of stem cell marker genes (including SOX9, LGR5, CD44, AXIN2, and CDH2). Furthermore, organoids cultured without EphrinA1-Fc showed significantly reduced expression of these genes. Together, these results demonstrate a functional role for EphrinA1/EphA1 signaling in regulating the stem cell properties of the organoids (Figure 2H).

### Formation and characterization of endometrial luminal epithelial organoids with apical-out polarity

**Figure 3:**
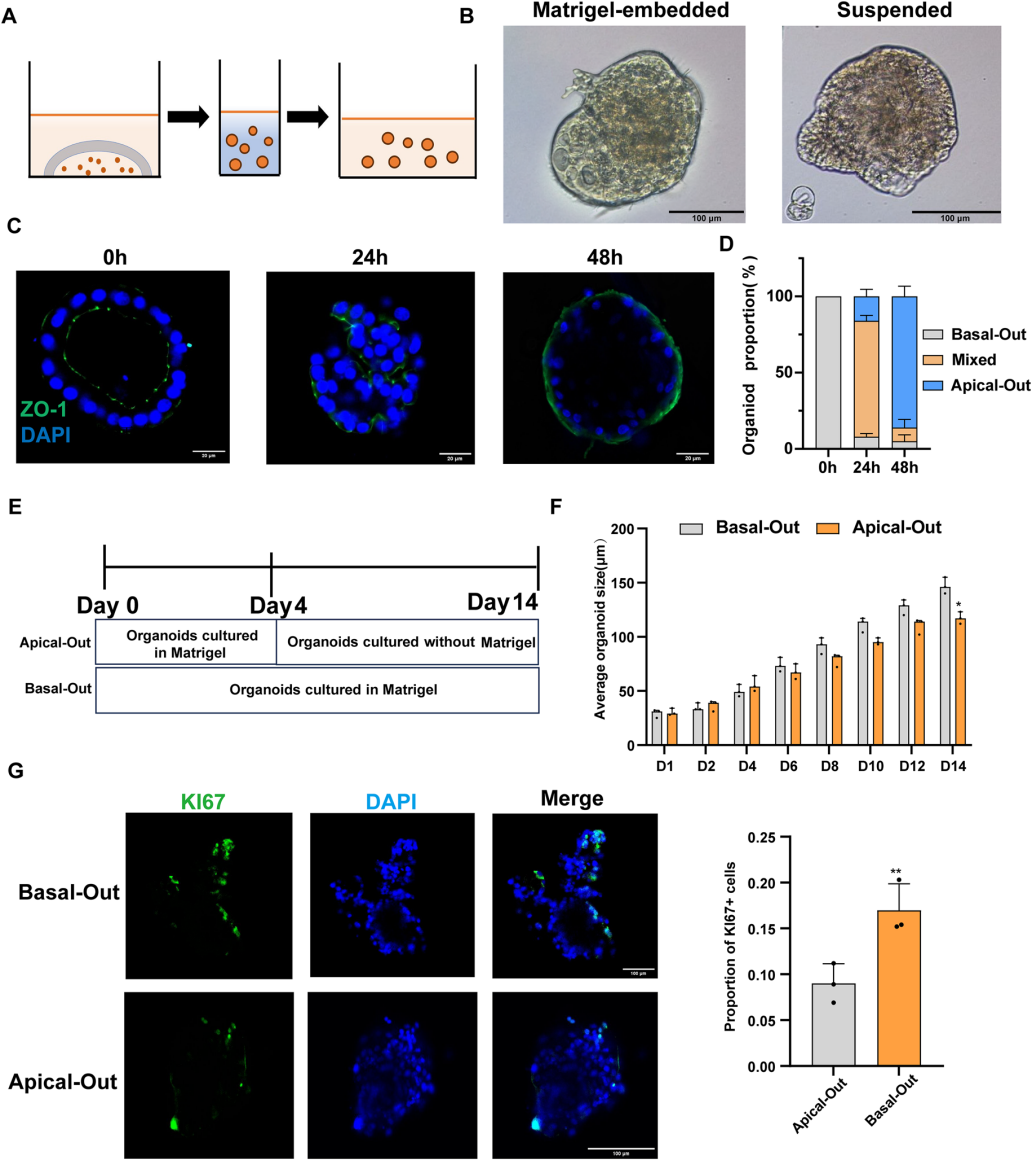
**Formation and characterization of endometrial luminal epithelial organoids with apical-out polarity. A** Schematic illustration of the protocol for establishing apical-out polarity. Organoids were released from Matrigel and transferred to ultralow-attachment plates for suspension culture to induce polarity reversal. **B** Morphological comparison after 48 h of suspension culture. Reversed organoids exhibit a distinct outer boundary and a translucent central lumen, in contrast to the basolateral-out (non-reversed) controls. **C** Representative immunofluorescence images depicting the relocalization of the tight junction protein zonula occludens-1 (ZO-1) during polarity reversal. **D** Quantitative analysis of the proportion of different organoid types (apical-out, basolateral-out, and indeterminate) over the course of polarity reversal. Each data point represents the value from one biological replicate (i.e., one independent experiment with organoids derived from one ewe). Data are presented as median with interquartile range of *N* = 3 biological replicates. **E** Schematic workflow for assessing the proliferative capacity of the two organoid polarity types (apical-out versus basal-out). **F** Longitudinal measurement of organoid diameter under the conditions outlined in **E**. Each data point represents the mean diameter (calculated from ≥ 30 organoids) for one biological replicate. Data are presented as median with interquartile range of *N* = 3 biological replicates. **G** Proliferation analysis via Ki67 immunofluorescence (green) in day 14 organoids. Nuclei are counterstained with DAPI (blue). Scale bars: **B** 20 µm; **C**, **G** 100 µm. Representative images from organoids derived from three individual ewes (*N* = 3).

During conventional 3D Matrigel culture, ovine endometrial luminal epithelial organoids develop with basal-out polarity, where the apical membrane faces the internal lumen. To establish an apical-out configuration that better mimics the in vivo orientation of luminal epithelium, organoids were released from Matrigel and transferred to suspension culture using low-attachment plates (Figure 3A). Following 48 h in suspension, reversed organoids exhibited a clearly defined outer boundary and distinct epithelial morphology, characteristics not observed in non-reversed controls (Figure 3B). The dynamics of polarity reversal were monitored via immunofluorescence localization of the tight junction protein zonula occludens-1 (ZO-1). After 24 h of suspension culture, 76% of organoids showed partial polarity reversal, displaying mixed apical-in and apical-out characteristics. By 48 h, 86% of organoids had fully reversed to a consistent apical-out polarity (Figures 3C, D). To assess the functional impact of polarity orientation on proliferative capacity, organoid size and proliferation markers were systematically evaluated (Figure 3E). Following Matrigel release on day 4, apical-out organoids displayed significantly reduced average diameter compared with basal-out controls from day 10 onward (*p* < 0.05), with this size difference maintained throughout the subsequent culture period (Figure 3F). Consistent with these findings, quantification of Ki-67-positive cells on day 14 revealed significantly lower proliferation rates in apical-out organoids compared with basal-out organoids (*p* < 0.05) (Figure 3G). These results demonstrate that the apical-out polarity state is associated with markedly reduced proliferative activity in endometrial luminal epithelial organoids.

### Hormone responsiveness in apical-out and basal-out organoids

**Figure 4:**
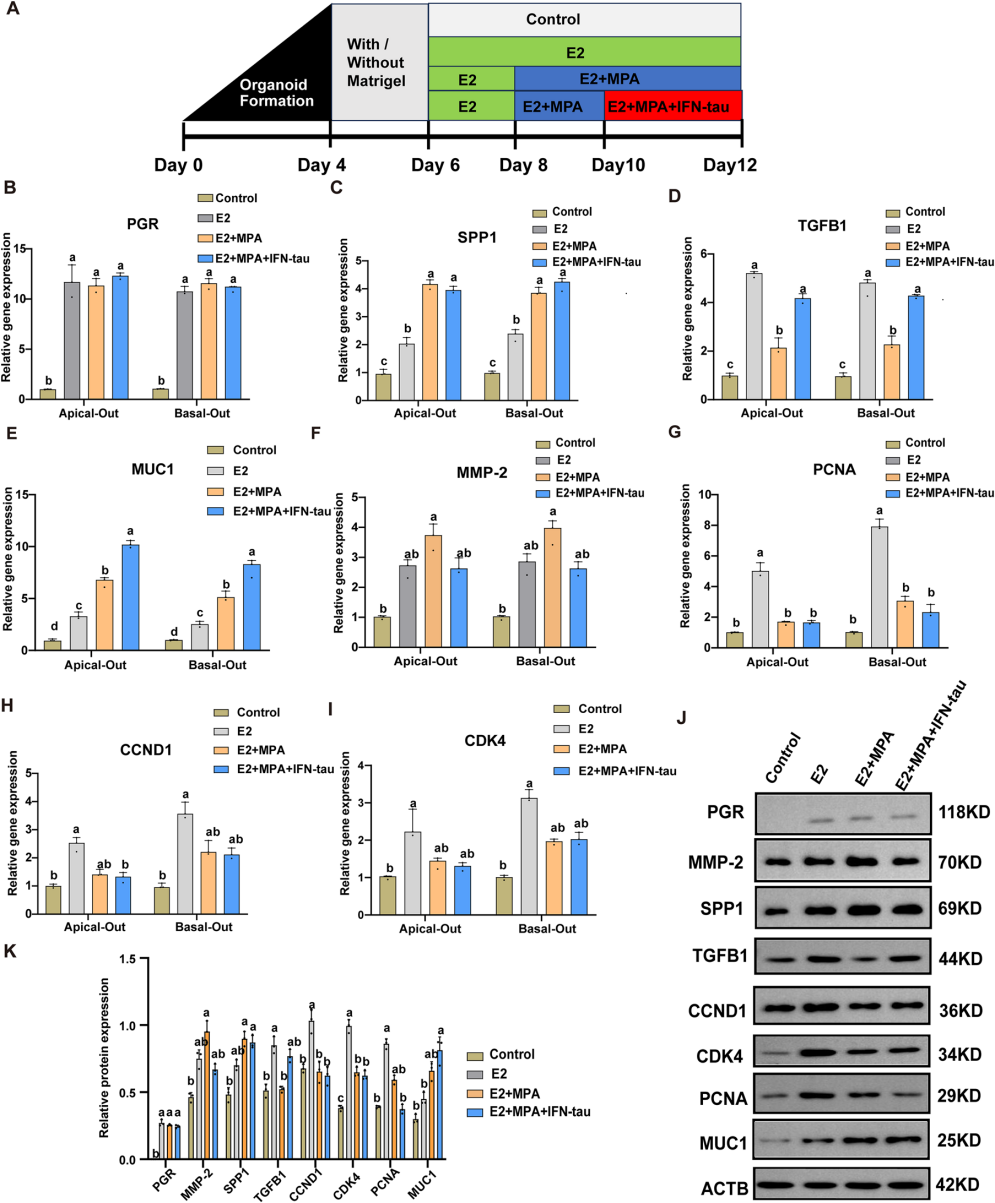
**Apical-out organoids retain core hormonal responsiveness, equivalent to conventional cultures. A** Schematic of the experimental design. Organoids were formed (days 0–4) and flipped (days 4–6) to generate apical-out organoids. From day 6 to 12, both basal-out and apical-out organoids were treated with different hormones as indicated. **B**–**I** Relative mRNA expression levels of the indicated genes (PGR, progesterone receptor; SPP1, secreted phosphoprotein 1; TGFB1, transforming growth factor beta 1; MUC1, mucin 1; MMP2, matrix metallopeptidase 2; PCNA, proliferating cell nuclear antigen; CCND1, cyclin D1; CDK4, cyclin-dependent kinase 4) in hormone-treated organoids. **J**, **K** Relative protein levels of the indicated markers in hormone-treated apical-out organoids, as determined by western blot. For all quantitative panels **B**–**K**, each data point represents one independent biological replicate (an organoid line derived from one ewe; *N* = 3). Data across the three biological replicates are presented as median with interquartile range. Statistically significant differences among treatment groups were determined using the Kruskal–Wallis test, followed by Dunn’s post hoc test for multiple comparisons. Different superscript letters (a–d) denote statistically significant differences (*p* < 0.05).

To determine whether polarity orientation affects hormonal response, both apical-out and basal-out organoids were subjected to a physiologically relevant hormonal regimen: E2, E2 + MPA, or E2 + MPA + IFN-tau (Figure 4A). Quantitative RT-PCR analysis revealed that both organoid types exhibited nearly identical transcriptional responses across all treatment conditions. Key receptivity-related genes displayed characteristic hormonal regulation: *PGR* expression was specifically upregulated by E2; *SPP1* showed robust induction by E2 with further enhancement upon MPA addition; *TGFB1* exhibited a triphasic pattern of E2-induced activation, MPA-mediated suppression, and IFN-tau-dependent rescue; while *MUC1* demonstrated progressive upregulation across treatment stages (Figures 4B–E). Matrix remodeling and proliferation-associated genes also showed consistent responses between polarity models, with *MMP-2* peaking under E2 + MPA but attenuated by IFN-tau, and proliferation markers (*PCNA*, *CCND1*, *CDK4*) being induced by E2 yet suppressed by MPA co-treatment (Figures 4F–I). At the protein level, the expression changes for the majority of the examined markers aligned with their mRNA profiles in apical-out organoids, confirming the general concordance between transcriptional and translational responses (Figures 4J, K). The full, uncropped Western blot membranes are provided in Additional file 8. Collectively, these comprehensive analyses demonstrate that apical-out organoids retain full hormonal response capability, with gene expression profiles equivalent to those of conventional basal-out cultures.

### Apical-out organoids transcriptionally recapitulate luminal epithelium

**Figure 5:**
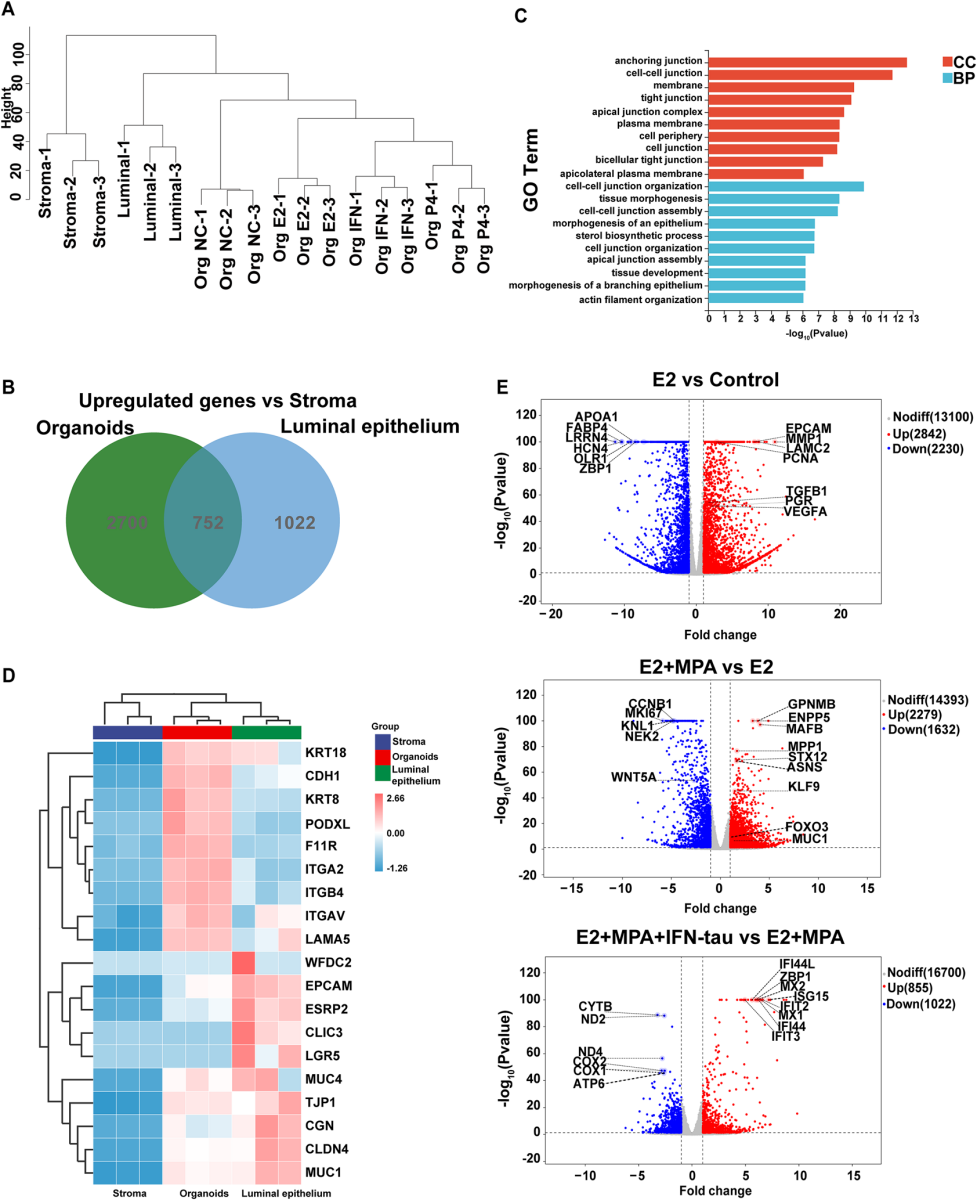
**Luminal epithelial organoids closely mimic the transcriptomic profile of native endometrial luminal epithelium. A** Hierarchical clustering of 3996 differentially expressed genes between native luminal epithelium and stromal cells, identified by DESeq2 with thresholds of |log_2_ fold change|≥ 1 and adjusted *p* ≤ 0.01. Clustering (using Euclidean distance and complete linkage) demonstrates transcriptional similarity between organoids and native luminal epithelium, while stromal cells form a distinct cluster. **B** Venn diagram identifies 752 co-upregulated genes (fold change ≥ 2, adjusted *p* ≤ 0.01) shared between luminal epithelium and organoids relative to stromal cells. **C** Gene Ontology (GO) analysis of the 752 co-upregulated genes was performed using TOPGO (version 2.50.0) with Benjamini–Hochberg correction. The top 10 significantly enriched biological processes (adjusted *p* ≤ 0.05) are shown, predominantly associated with epithelial functions. **D** Heatmap of RNA-seq expression levels for selected epithelial marker genes in native luminal epithelial cells, stromal cells, and organoids. The color key represents row-scaled expression *Z*-scores, with red indicating high expression and blue indicating low expression. Expression patterns of general epithelial markers (cadherin 1 [CDH1], keratin 18 [KRT18], and epithelial cell adhesion molecule [EpCAM]), a mucosal secretory marker (mucin 1 [MUC1]), and ovine-specific luminal markers (WAP four-disulfide core domain 2 [WFDC2] and insulin-like growth factor binding protein 2 [IGFBP2]) demonstrate transcriptional similarity between organoids and native epithelium. **E** Heatmap of differentially expressed genes in endometrial organoids under distinct hormonal treatments: estradiol (E2), Medroxyprogesterone acetate (MPA), and E2 + MPA combined. Data are derived from *N* = 3 independent organoid lines, each established from a different ewe.

To evaluate the transcriptional fidelity of the organoid model, we compared the bulk RNA-seq profiles of apical-out organoids with reference transcriptomes of native luminal epithelial and stromal cells (see “Methods” and Additional file 2). Differential expression analysis between the native cell types identified 3996 significantly differentially expressed genes (Additional file 3). Hierarchical clustering based on these genes demonstrated that organoids grouped closely with native luminal epithelial cells, while stromal cells formed a distinct, separate cluster (Figure 5A).

Comparative transcriptomic analysis identified 752 genes that were co-upregulated (fold change ≥ 2, *p* ≤ 0.01) in both native luminal epithelium and organoids relative to stromal cells (Figure 5B). Gene Ontology enrichment analysis of these shared genes revealed significant associations with epithelial-specific biological processes, including cell–cell adhesion, epithelial development, and secretion (Figure 5C). Expression validation of key epithelial markers confirmed that organoids strongly expressed general epithelial markers (CDH1, KRT18, and EpCAM), mucosal secretory markers (MUC1), and established ovine-specific luminal epithelial markers (WFDC2 and IGFBP2), mirroring the expression pattern observed in native luminal epithelial cells (Figure 5D).

Furthermore, transcriptome analysis under hormonal stimulation demonstrated that organoids exhibited physiologically relevant responses: E2 treatment upregulated estrogen-responsive genes, E2 + MPA treatment induced progesterone-activated genes, and E2 + MPA + IFN-tau treatment robustly activated classical interferon-stimulated genes (Figure 5E). Collectively, these results demonstrate at the transcriptomic level that the luminal epithelial organoids faithfully recapitulate the characteristics of their native counterpart and maintain appropriate hormonal response capabilities.

### Multidimensional functional analysis of endometrial organoids: effects on blastocysts, trophoblast proliferation, and substrate-specific adhesion

**Figure 6:**
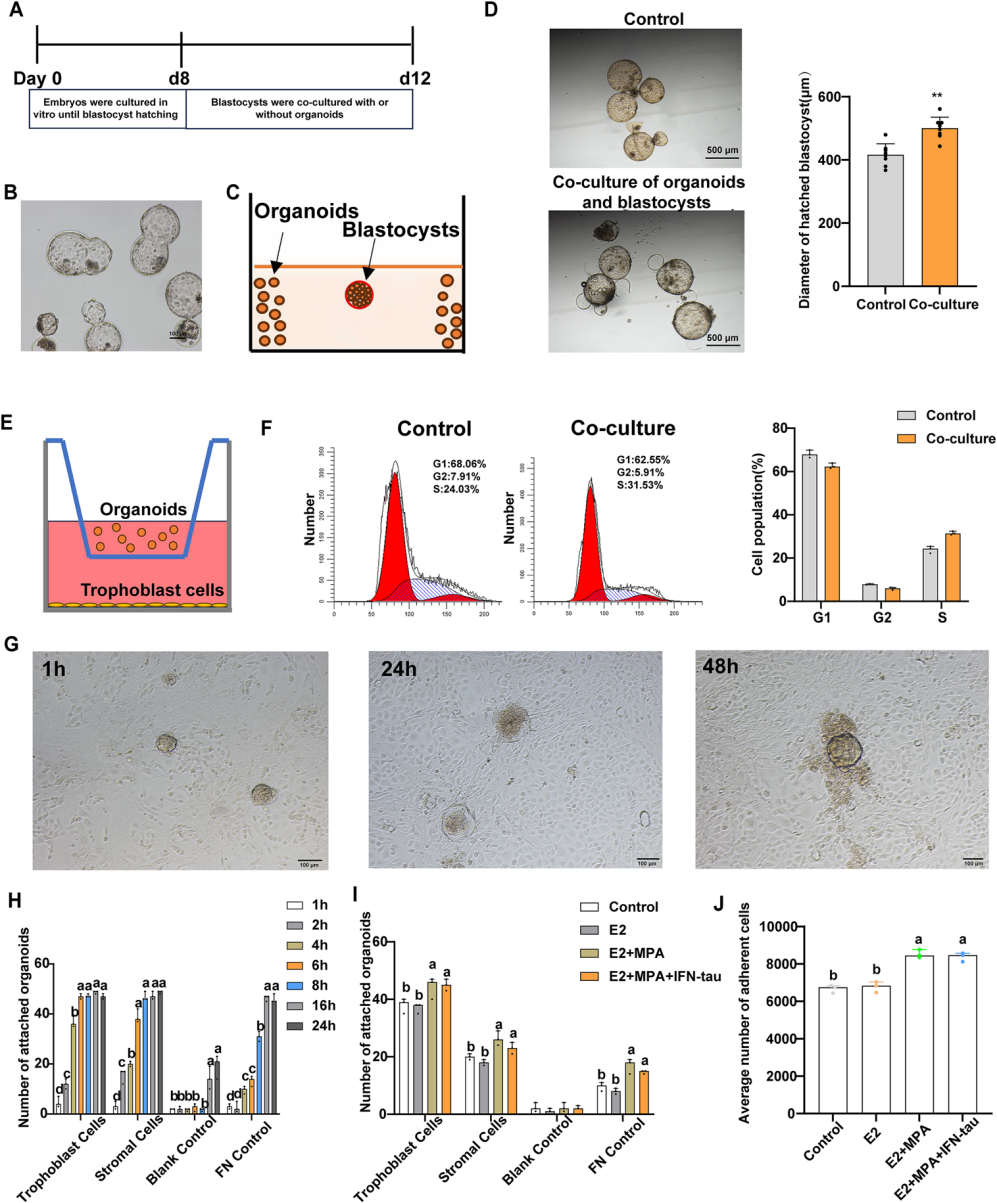
**Multidimensional functional analysis of endometrial organoids: effects on blastocysts, trophoblast proliferation, and substrate-specific adhesion. A** Schematic of the blastocyst–organoid co-culture system. **B** Blastocysts cultured to the hatching stage in the same dish (day 8). A single hatching blastocyst was then used for co-culture with organoids. **C** Design of the noncontact co-culture model: blastocysts and apical-out organoids shared medium in the same well without direct physical contact for 4 days. **D** Bright-field images showing blastocyst morphology and quantification of blastocyst diameter following the 4-day noncontact co-culture. **E** Schematic of the Transwell-based indirect co-culture system: organoids and trophoblast cells were separated by a permeable membrane to allow paracrine signaling. **F** Cell cycle distribution of trophoblast cells following a 48-h indirect co-culture with organoids using the Transwell system. **G** Adhesion dynamics between conventional (basolateral-out) organoids and trophoblast cells, shown at different time points (e.g., 1, 2, and 4 h). **H** Time to complete organoid adhesion on different substrates: trophoblast monolayers, endometrial stromal cell monolayers, fibronectin-coated dishes, and standard culture plates. **I** Adhesion efficiency at 4 h of organoids pretreated with Control, E2, E2 + MPA, or E2 + MPA + IFN-tau on stromal cell monolayers, trophoblast cell monolayers, and fibronectin-coated dishes. **J** Adherent cell number (OD450) on fibronectin after 4 h for organoids pretreated with Control, E2, E2 + MPA, or E2 + MPA + IFN-tau. For quantitative panels: Data in panel **D** are from *N* = 8 independent blastocysts. Data in panels **F**, **H**, **I**, and **J** are from *N* = 3 independent biological replicates (each data point represents one organoid line derived from a different ewe). All data are presented as median with interquartile range. Statistically significant differences across multiple groups were assessed using the Kruskal–Wallis test, followed by Dunn’s post hoc test; different superscript letters (a–d) denote *p* < 0.05.

A blastocyst–organoid co-culture system was established to evaluate the functional role of endometrial organoids in embryo development (Figure 6A). Hatched day 8 blastocysts (Figure 6B) were co-cultured with apical-out organoids using a noncontact model where both components shared the same medium without direct physical contact (Figure 6C). After 4 days of co-culture, blastocysts maintained normal morphology and showed a significant 20.25% increase in average diameter compared with controls (Figure 6D), suggesting that secretory factors from organoids support embryonic development.

To determine whether this embryonic enlargement resulted from trophoblast proliferation rather than fluid accumulation, a Transwell-based indirect co-culture system was employed, separating organoids and trophoblast cells with a permeable membrane to prevent direct contact while allowing molecular exchange (Figure 6E). The S-phase proportion of trophoblast cells showed a nonsignificant increasing trend after 2-day co-culture (Figure 6F), suggesting a possible pro-proliferative effect of organoid-secreted factors that requires further investigation. Representative images of organoids adhering to trophoblast cells at 1, 24, and 48 h are shown in Figure 6G. Substantial morphological restructuring of the organoids was observed following adhesion to the trophoblast monolayer. Analysis of adhesion dynamics revealed substantial substrate-dependent variation. Organoids adhered most rapidly to trophoblast monolayers (complete within 6 h), followed by endometrial stromal cells (8 h). Adhesion was markedly delayed on fibronectin-coated dishes (requiring 16 h for completion), and most sluggish on standard culture plates, where adhesion was barely initiated by 16 h (Figure 6H). Hormonal pretreatment significantly enhanced adhesion kinetics to various extracellular matrices within 4 h (Figure 6I). This enhanced adhesion capacity was quantitatively confirmed using cell viability assays, where organoids were dissociated into single cells and allowed to adhere to fibronectin-coated surfaces for 4 h. The E2 + MPA and E2 + MPA + IFN-tau treated groups exhibited the highest OD450 values, indicating the greatest number of adherent cells (Figure 6J). Collectively, these results demonstrate that endometrial organoids functionally support blastocyst development through promoting trophoblast proliferation and exhibit substrate-specific adhesion capacity that is further enhanced by hormonal stimulation.

## Discussion

As organoid technology becomes increasingly prevalent in reproductive biology studies, researchers have successfully developed endometrial organoid models across multiple species, enabling precise simulation of intricate in vivo microenvironments and cellular dynamic [37, 38]. Compared with mice and humans, ruminants exhibit a more complex uterine structure and species-specific cyclic regulatory mechanisms. Therefore, the development of a sheep endometrial organoid model not only holds significant theoretical importance but also provides a powerful tool for investigating reproductive regulation mechanisms in ruminant species [39, 40].

This study focuses on the luminal epithelium (LE) of the endometrium, a critical cell population that plays a pivotal role during embryo implantation. The LE is functionally specialized for embryo recognition, signal sensing, and initial adhesion, making it indispensable for successful pregnancy establishment [41, 42]. A luminal epithelium-enriched cell population was isolated from ovine endometrial caruncle tissue by mechanical dissociation and enzymatic digestion. This isolated population was then used to develop a stable organoid culture system within a three-dimensional Matrigel matrix. During culture optimization, we demonstrated that the combination of WNT3A and CHIR99021 synergistically promotes organoid formation efficiency. WNT3A, a canonical WNT pathway ligand, activates β-catenin-dependent transcriptional regulation, while CHIR99021, a potent GSK3β inhibitor, synergistically enhances downstream WNT signaling activity [43, 44]. We observed that WNT3A alone yielded suboptimal organoid formation efficiency, whereas combinatorial treatment with CHIR99021 significantly increased both organoid number and diameter. These findings align with the WNT-dependent characteristics observed in human and murine endometrial organoid models, yet reveal a stronger synergistic pathway dependence in ovine systems. This suggests that ovine luminal epithelium may possess a higher activation threshold for WNT signaling [23, 26]. Notably, the combined application of Y-27632 and SB202190 significantly enhanced both the adult stemness and proliferative capacity of luminal epithelial cells. This represents a technical innovation in our culture system optimization.

Intriguingly, we observed a similar phenomenon during the development of ovine ruminal epithelial organoids, indicating a conserved role of these two factors in maintaining stemness properties across different ovine epithelial lineages [45]. Notably, our study represents the first attempt to incorporate the EphA1 ligand (EphrinA1) as a modulator, revealing its critical role in both promoting organoid formation and maintaining cellular polarity. EphA1, a membrane-bound tyrosine kinase receptor, plays multifaceted roles in epithelial cell organization, intercellular communication, and tissue boundary maintenance. Notably, this receptor has been demonstrated to promote proliferative activity in bovine endometrial epithelial cells [46, 47]. Supplementation of the organoid culture system with the EphA1 ligand, EphrinA1-Fc, resulted in significantly increased organoid diameter, indicating that activation of the EphA1 receptor potently promotes organoid proliferation and development. These findings provide the first evidence for the functional role of EphrinA1/EphA1 ligand-receptor signaling in uterine organoid formation, paving the way for future investigations into its mechanistic involvement in embryo implantation-related tissue remodeling. The successful development of ovine endometrial luminal epithelium organoids thus hinges on the coordinated regulation of multiple signaling pathways. Our systematic optimization demonstrates that: (1) WNT3A/CHIR99021 maintains stemness properties, (2) Y-27632/SB202190 enhances cellular viability and stability, and (3) activation of EphA1 signaling by its ligand EphrinA1 mediates proliferative activity. This refined culture system not only provides a technical platform for robust reconstruction of endometrial luminal architecture, but also establishes a critical foundation for modeling the embryo–uterine dialog microenvironment.

During the development of ovine endometrial luminal epithelium organoids, evaluating their structural and phenotypic recapitulation is critical for validating the physiological relevance of the organoid model [48]. Through comprehensive histological staining, ultrastructural analysis, and phenotypic marker characterization, we systematically validated the ability of organoids to recapitulate key features of luminal epithelium. At the structural level, organoids developed vesicle-like cavities and exhibited mucin secretory activity—hallmarks of polarized epithelial differentiation. These findings collectively demonstrate the organoids’ functional competence in mimicking the uterine luminal microenvironment. Glycans and mucins constitute essential secretory components of the endometrium that critically support embryonic development and implantation [49]. Notably, PAS-positive staining confirmed the secretion of glycoprotein-like substances by the organoids, demonstrating their ability to recapitulate the endocrine functionality of the native endometrium. Furthermore, microvilli serve as critical sensory and regulatory structures for epithelial cells to perceive and adapt to their microenvironment [49]. Transmission electron microscopy (TEM) revealed well-developed microvilli and secretory vesicles, providing ultrastructural evidence for the functional maturation of differentiated epithelium in these organoids. Phenotypic characterization revealed consistent expression of luminal epithelial markers (KRT18, TROP2, and EpCAM) in the organoids, confirming their tissue origin and recapitulation of in vivo expression patterns [50]. However, the organoids currently lack a fully developed basement membrane and stromal cell support, which may compromise their long-term culture stability and maturation. To address this limitation, future studies could integrate co-culture systems or biomechanical stimulation to enhance structural complexity and functional fidelity [48, 51].

In vivo, embryo implantation requires direct contact between endometrial luminal epithelium and trophoblasts. Conventional epithelial organoids exhibit an “apical-in” polarity (apical surface facing the lumen, basolateral side outward), which limits their ability to fully simulate the intrauterine environment, particularly for studying physiological processes involving luminal interactions [52]. To address this limitation, we developed an apical-out polarity reversal model, inspired by intestinal and murine endometrial organoid systems [53, 54], where the apical surface is exposed to the culture medium to better mimic the natural uterine luminal interface. During polarity reversal, organoids exhibited slight flattening with reduced or absent luminal structures, likely due to reorganization of cell junctions and cytoskeletal arrangements. The polarity reversal was completed within approximately 48 h—significantly faster than observed in murine intestinal organoids, possibly owing to their larger luminal cavities requiring more time for structural reorganization. Notably, suspended apical-out organoids showed significantly reduced size compared with Matrigel-embedded cultures after day 8. Ki-67 staining revealed moderately decreased proliferative activity in reversed organoids, suggesting that polarity alteration may impose transient proliferative suppression. This phenotypic shift may stem from cytoskeletal remodeling during polarity reversal and loss of cell–matrix interactions, warranting further investigation into its mechanistic basis and functional implications.

The endometrial function is critically regulated by the cyclic actions of reproductive hormones, particularly estradiol (E2) and progesterone (P4), which determine uterine receptivity and the opening/closing of the implantation window [55]. Therefore, evaluating the hormonal responsiveness of organoid models serves as a crucial indicator of their physiological relevance and in vivo mimicry capability. Through systematic analysis of the response characteristics to reproductive hormones and IFN-tau in both apical-out and basal-out organoids, our study provides profound insights into the molecular regulatory mechanisms of endometrial epithelial cells under different polarity states. Despite their distinct morphological differences, we made the striking observation that these two polarity-variant organoid models exhibited highly consistent gene expression profiles upon hormonal and IFN-tau stimulation. This significant finding provides an important foundation for applying organoid models in reproductive biology research. The results clearly demonstrate the central role of E2 in regulating endometrial receptivity. The specific response of PGR to E2 (Figure 4B) aligns with established estrogen regulatory mechanisms [56]. Notably, the expression patterns of SPP1 and MUC1 (Figures 4C, E) faithfully recapitulate the molecular signatures of epithelial cells during the implantation window in vivo. The synergistic enhancement of SPP1 by MPA is particularly striking, underscoring progesterone’s unique role in embryo adhesion [57]. Interestingly, the E2-induced upregulation of *SPP1* in our luminal epithelial organoids contrasts with established in vivo findings in sheep, where estrogen does not induce *SPP1* in glandular epithelium [58]. This divergence underscores fundamental differences in hormonal regulation between endometrial epithelial compartments. Moreover, while *SPP1* is primarily progesterone-dependent in the ovine endometrium, its estrogen responsiveness is conserved in the luminal epithelium of pigs and mice [59]. Thus, our organoid model reveals a latent, lineage-specific estrogen signaling pathway in ovine luminal epithelium that is likely masked by the dominant progesterone signaling and complex tissue context in vivo. The dynamic regulation of TGFB1 (Figure 4D) further reveals the intricate crosstalk between hormonal and embryonic signals. The reversal of MPA’s inhibitory effect by IFN-tau highlights its critical function in maintaining immune homeostasis at the maternal–fetal interface. This pattern closely mirrors the hormonal response of endometrial epithelial cells in vivo, confirming that the organoid system effectively mimics the physiological hormonal milieu [10, 60].

The expression changes in proliferation-related genes (Figures 4G–I) accurately replicate the proliferation-differentiation switch observed in the endometrium during embryo implantation. The pro-proliferative effect of E2 contrasts sharply with the suppressive role of MPA, while the “neutral” response to IFN-tau aligns with its known immunomodulatory function [61, 62]. The regulation of *MMP-2* (Figure 4F) is particularly noteworthy—its peak expression under E2 + MPA treatment and subsequent IFN-tau-mediated suppression likely reflect the embryo’s precise control over stromal remodeling. Western blot analysis (Figures 4J, K) further validates these findings at the protein level, reinforcing the robustness of our conclusions. Although apical-out organoids exhibit slightly reduced proliferative capacity, their consistent hormonal responsiveness makes them an ideal model for studying the uterine luminal microenvironment. Our study not only confirms the reliability of organoids in simulating endometrial physiology but, more importantly, reveals that polarity has a limited impact on epithelial cell functional regulation. These findings establish a solid foundation for future research on maternal–fetal crosstalk using apical-out organoids and provide key insights for developing more physiologically relevant in vitro models.

To further validate the molecular-level recapitulation of luminal epithelium by our engineered organoids, we performed an integrative analysis comparing their transcriptomic profiles with bulk RNA-seq (BULK-seq) data generated from endometrial single-cell sequencing. Cluster analysis demonstrated that the overall transcriptional signature of the organoids exhibited greater similarity to luminal epithelium than to stromal cells, indicating successful preservation of luminal epithelial transcriptional characteristics during in vitro culture. These findings are consistent with previous observations in both human and murine uterine organoid studies, confirming that organoids can maintain stable epithelial lineage expression independent of stromal cell support [25, 26]. This comparative transcriptomic analysis provides compelling evidence that our organoid model faithfully captures the essential molecular attributes of native luminal epithelium.

Differential expression analysis further revealed that the organoids and luminal epithelium shared high expression levels of multiple epithelial marker genes (including CDH1, KRT18, and EpCAM), as well as pregnancy-specific functional molecules WFDC2 and IGFBP2. These findings not only demonstrate the model’s fidelity in maintaining cellular lineage identity but also suggest its potential secretory functionality. Notably, the epithelial secretory capacity plays a crucial role in supporting early embryonic development by producing glycoproteins, cytokines, and chemokines—processes whose regulation fundamentally depends on the organoid system’s ability to faithfully recapitulate hormonal and signaling pathway responses [7, 63].

In terms of hormonal responsiveness, the organoids demonstrated characteristic upregulation of key downstream genes (including PCNA, PGR, and KLF9) following estrogen and progesterone treatment, effectively mimicking the hormone-sensitive features of endometrium during either the menstrual cycle or early pregnancy. Particularly noteworthy was the robust upregulation of interferon pathway effectors (MX2, ISG15, and IFIT3) under combined E2 + MPA + IFN-tau stimulation, which precisely recapitulates the maternal immune response during embryonic recognition observed in vivo [10]. These results collectively demonstrate the organoid system’s dual competence in responding to both reproductive endocrine signals and embryonic cues, thereby establishing a biologically relevant in vitro platform for modeling the implantation window. Building upon these findings, we further investigated the potential of endometrial organoids to recapitulate key aspects of maternal–embryo crosstalk during early implantation. Co-culture experiments revealed that the organoids significantly enhanced in vitro growth of day-8 blastocysts, suggesting their capacity to secrete embryotrophic factors that promote proliferation and survival. While the N2 + B27 culture system has been shown to support embryonic proliferation in vitro, it fails to fully mimic the rapid blastocyst elongation observed in vivo [64]. To address this limitation, we supplemented the N2/B27 medium with additional factors known to enhance luminal epithelial activity. This modified formulation supported limited blastocyst expansion even in the absence of organoids, but co-culture with organoids resulted in significantly accelerated growth kinetics (*p* < 0.01). The observed increase in blastocyst volume could originate from either trophectoderm or inner cell mass proliferation—a distinction requiring future lineage-specific marker analysis. Notably, recent metabolomic studies have identified multiple critical metabolites in organoid-conditioned media [22]. Our developed co-culture system, when combined with such multi-omics approaches (e.g., metabolomics and proteomics), will enable systematic identification of key embryotrophic factors. This strategy promises to bridge the current gap between in vitro models and the complex embryonic development occurring in utero. While conventional in vitro models for studying maternal–fetal interactions in ruminants have primarily relied on two-dimensional (2D) co-culture systems of endometrial epithelial cells and trophoblasts [65, 66], the present study developed an advanced three-dimensional model using apical-out endometrial luminal epithelial organoids co-cultured with trophoblast cells. Our results demonstrated that the organoids established stable adhesions with trophoblasts within remarkably short time periods, while maintaining consistent adhesive capacity across multiple passages—indicating excellent experimental reproducibility and translational potential of this model. Notably, reproductive hormones significantly enhanced organoid–trophoblast adhesion, with the E2 + MPA and E2 + MPA + IFN-tau treatment groups achieving particularly high attachment ratios in minimal time. This hormone-responsive pattern closely recapitulates the enhanced endometrial–trophoblast interactions observed during in vivo pregnancy under hormonal regulation [15], thereby confirming the superior physiological relevance of our ovine luminal epithelial organoid system for modeling hormone-mediated uterus–embryo crosstalk.

While our in vitro system effectively assesses the adhesive capacity of endometrial luminal epithelial organoids, it primarily captures physical attachment rather than the complex cellular fusion events occurring during in vivo implantation [67]. This simplified model, focusing on organoid–matrix interactions, fundamentally differs from the orchestrated in vivo process that involves dynamic crosstalk among the epithelium, stroma, immune cells, and extracellular matrix [68, 69]. Nevertheless, the key advantage of our approach lies in its ability to isolate the intrinsic adhesive properties of the luminal epithelium. The enhanced adhesion following E2 + MPA pretreatment—recapitulating the hormonal priming essential for uterine receptivity in vivo—highlights the physiological relevance of our system. Thus, although not fully replicating implantation complexity, this reductionist platform offers a valuable tool for delineating the specific contribution of the receptive luminal epithelium, which remains challenging to study in intact organisms. To further resolve the cellular dynamics underlying the adhesion events observed here, future studies employing live-cell imaging with lineage-specific fluorescent markers will be essential to visually dissect the spatiotemporal sequence of initial contact and cytoskeletal rearrangement. This approach will help bridge our physical attachment metrics with the complex adhesion-to-fusion continuum that occurs in vivo.

In summary, this study demonstrates that our newly developed ovine endometrial organoids faithfully mimic the native luminal epithelium in structure and secretion, while functionally enhancing embryonic development and trophoblast adhesion. However, several important limitations remain: (1) the absence of stromal cells, immune cells, and other functional cell types in the culture system deprives luminal epithelial cells of critical paracrine signaling from these cellular components, thus failing to fully mimic the in vivo microenvironment; (2) while responsive to E2, MPA, and IFN-tau, the static hormone concentrations and timing in vitro cannot precisely replicate the dynamic fluctuations during estrous cycles; (3) although supporting blastocyst proliferation and trophoblast adhesion, the proliferation patterns differ substantially from in vivo conditions, as evidenced by the markedly slower conceptus elongation in vitro, making complete simulation of ovine blastocyst–endometrium adhesion exceptionally challenging. Future studies should integrate microfluidic devices, co-culture matrix systems, and 3D bioprinting technologies to develop more physiologically relevant maternal–fetal interface models with enhanced complexity, thereby providing an improved foundation for reconstructing ruminant uterus–embryo interactions that better approximate in vivo conditions [70, 71].

## Conclusions

This study successfully established functional ovine endometrial luminal epithelial organoids and systematically validated their morphological, molecular, and functional similarities to native tissue. The developed model faithfully recapitulates characteristic luminal epithelial features—including polarized architecture, mucin secretion, and specific molecular marker expression—while accurately simulating key physiological processes such as hormonal response and embryo interaction. Transcriptomic analysis confirmed the high similarity between organoids and native luminal epithelium. Functional assays further demonstrated the organoids’ ability to support embryonic development and promote trophoblast proliferation. Moreover, this study revealed a novel role of EphA signaling in regulating stemness properties in organoids. This model provides a reliable platform for investigating embryo implantation mechanisms in ruminants, lays a solid foundation for developing new strategies to improve livestock reproductive efficiency, and offers new technical approaches for studying reproductive disorders.

## Supplementary Information

**Additional file 1 Raw RNA-seq counts of ovine endometrial luminal epithelial organoids**. This dataset contains the raw read counts for all organoid samples used in this study, as deposited in the CNGB Sequence Archive under accession no. CNP0008511.

**Additional file 2 Pseudo-bulk luminal epithelial reference transcriptome**. This dataset contains the aggregated pseudo-bulk transcriptomic profile of native luminal epithelial cells, generated from scRNA-seq data of endometrial tissues (accession no. PRJCA056151). This file served as the native LE reference for correlation analysis with organoid bulk RNA-seq data.

**Additional file 3 Differentially expressed genes between native cell types**. This dataset lists the 3996 significantly differentially expressed genes identified between native luminal epithelial cells and stromal cells (|log_2_FC| ≥ 1, adjusted *p* < 0.05), which were used for hierarchical clustering analysis in Figure 5A.

**Additional file 4 Negative controls for immunofluorescence staining in organoids.**

**Additional file 5 Quality assessment of total RNA by microcapillary electrophoresis in organoids under different treatments.** RIN > 9.0.

**Additional file 6 Immunohistochemical identification of ovine trophoblast cells (positive for CK-7, GATA3, and FABP3)**.

**Additional file 7 Immunofluorescence staining of FOXA2 and corresponding FPKM values from transcriptome sequencing in ovine organoids.**

**Additional file 8 Original uncropped images of all Western blot results.**

**Additional file 9 Ct values from no-reverse transcription controls (−RT) and experimental cDNA groups for all target genes.**

## Data Availability

The datasets used and analyzed during the current study are available from the corresponding author on reasonable request.

## Abbreviations

Apical-out: apical-out polarity
AXIN2: Axin 2
Basal-Out: basal-out polarity
CDH1: cadherin 1
CDH2: cadherin 2
CD44: cluster of differentiation 44
Epha1: ephrin type-A receptor 1
EpCAM: epithelial cell adhesion molecule
ExM: organoid expansion medium
FGF10: fibroblast growth factor 10
FOXO3: forkhead box O3
FOXJ1: forkhead box J1
GO: Gene Ontology
H&E: hematoxylin and eosin
HGF: hepatocyte growth factor
IFN-tau: interferon-tau
IFIT3: interferon-induced protein with tetratricopeptide repeats 3
IGFBP2: insulin-like growth factor binding protein 2
ISG15: interferon-stimulated gene 15
Ki-67: marker of proliferation Ki-67
KLF9: Krüppel-like factor 9
KRT18: keratin 18
LE: luminal epithelium
LGR5: leucine-rich repeat-containing G-protein coupled receptor 5
LysoPC: lysophosphatidylcholine
MPA: medroxyprogesterone acetate
MMP-2: matrix metalloproteinase-2
MUC1: mucin 1
MX2: MX dynamin-like GTPase 2
NOG: Noggin
PAS: periodic acid–Schiff
PCNA: proliferating cell nuclear antigen
PEDV: porcine epidemic diarrhea virus
PGR: progesterone receptor
RSPO1: R-Spondin-1
SEM: standard error of the mean
SOX9: SRY-box transcription factor 9
SPP1: secreted phosphoprotein 1
TEM: transmission electron microscopy
TGFB1: transforming growth factor beta 1
TROP2: trophoblast cell surface antigen 2
VEGF: vascular endothelial growth factor
WFDC2: WAP four-disulfide core domain 2
WNT3A: Wnt family member 3A

## References

[1] Singh M, Chaudhry P, Asselin E (2011) Bridging endometrial receptivity and implantation: network of hormones, cytokines, and growth factors. J Endocrinol 210:5–14 21372150 10.1530/JOE-10-0461

[2] Robertson SA, Moldenhauer LM, Green ES, Care AS, Hull ML (2022) Immune determinants of endometrial receptivity: a biological perspective. Fertil Steril 117:1107–1120 35618356 10.1016/j.fertnstert.2022.04.023

[3] van der Weijden VA, Puntar B, Rudolf Vegas A, Milojevic V, Schanzenbach CI, Kowalewski MP, Drews B, Ulbrich SE (2019) Endometrial luminal epithelial cells sense embryo elongation in the roe deer independent of interferon-tau. Biol Reprod 101:882–892 31317179 10.1093/biolre/ioz129

[4] Quinn KE, Matson BC, Wetendorf M, Caron KM (2020) Pinopodes: recent advancements, current perspectives, and future directions. Mol Cell Endocrinol 501:110644 31738970 10.1016/j.mce.2019.110644 PMC6962535

[5] Lim W, Bae H, Bazer FW, Song G (2017) Functional roles of ephA-ephrin A1 system in endometrial luminal epithelial cells during early pregnancy. J Cell Physiol 232:1527–1538 27775163 10.1002/jcp.25659

[6] Zlotkowska A, Andronowska A (2019) Chemokines as the modulators of endometrial epithelial cells remodelling. Sci Rep 9:12968 31506569 10.1038/s41598-019-49502-5 PMC6736846

[7] Yoo I, Lee S, Cheon Y, Park TS, Ka H (2025) CD40 ligand and CD40: expression, regulation and function at the maternal-conceptus interface in pigs. Reproduction 169:e240396 39874127 10.1530/REP-24-0396

[8] Ng YH, Rome S, Jalabert A, Forterre A, Singh H, Hincks CL, Salamonsen LA (2013) Endometrial exosomes/microvesicles in the uterine microenvironment: a new paradigm for embryo-endometrial cross talk at implantation. PLoS ONE 8:e58502 23516492 10.1371/journal.pone.0058502 PMC3596344

[9] Yang Q, Liu J, Wang Y, Zhao W, Wang W, Cui J, Yang J, Yue Y, Zhang S, Chu M, Lyu Q, Ma L, Tang Y, Hu Y, Miao K, Zhao H, Tian J, An L (2022) A proteomic atlas of ligand-receptor interactions at the ovine maternal-fetal interface reveals the role of histone lactylation in uterine remodeling. J Biol Chem 298:101456 34861240 10.1016/j.jbc.2021.101456 PMC8733267

[10] Johnson GA, Bazer FW, Burghardt RC, Seo H, Wu G, Cain JW, Pohler KG (2024) The history of interferon-stimulated genes in pregnant cattle, sheep, and pigs. Reproduction 168:e240130 39028589 10.1530/REP-24-0130

[11] Johnson GA, Minela T, Seo H, Bazer FW, Burghardt RC, Wu G, Pohler KG, Stenhouse C, Cain JW, Seekford ZK, Soffa DR (2025) Conceptus elongation, implantation, and early placental development in species with central implantation: pigs, sheep, and cows. Biomolecules 15:1037 40723910 10.3390/biom15071037 PMC12293963

[12] Davenport KM, Ortega MS, Johnson GA, Seo H, Spencer TE (2023) Review: implantation and placentation in ruminants. Animal 17:100796 37567669 10.1016/j.animal.2023.100796

[13] Bazer FW, Wu G, Spencer TE, Johnson GA, Burghardt RC, Bayless K (2009) Novel pathways for implantation and establishment and maintenance of pregnancy in mammals. Mol Hum Reprod 16:135–152 19880575 10.1093/molehr/gap095 PMC2816171

[14] Spencer TE, Hansen TR (2015) Implantation and establishment of pregnancy in ruminants. Adv Anat Embryol Cell Biol 216:105 26450497 10.1007/978-3-319-15856-3_7

[15] Spencer TE, Johnson GA, Bazer FW, Burghardt RC (2004) Implantation mechanisms: insights from the sheep. Reproduction 128:657–668 15579583 10.1530/rep.1.00398

[16] Ahn J, Yoon MJ, Hong SH, Cha H, Lee D, Koo HS, Ko JE, Lee J, Oh S, Jeon NL, Kang YJ (2021) Three-dimensional microengineered vascularised endometrium-on-a-chip. Hum Reprod 36:2720–2731 34363466 10.1093/humrep/deab186 PMC8450871

[17] Eissa AM, Barros FSV, Vrljicak P, Brosens JJ, Cameron NR (2018) Enhanced differentiation potential of primary human endometrial cells cultured on 3D scaffolds. Biomacromol 19:3343–3350 10.1021/acs.biomac.8b00635 29928802

[18] Valentijn AJ, Saretzki G, Tempest N, Critchley HO, Hapangama DK (2015) Human endometrial epithelial telomerase is important for epithelial proliferation and glandular formation with potential implications in endometriosis. Hum Reprod 30:2816–2828 26498179 10.1093/humrep/dev267

[19] Classen-Linke I, Kusche M, Knauthe R, Beier HM (1997) Establishment of a human endometrial cell culture system and characterization of its polarized hormone responsive epithelial cells. Cell Tissue Res 287:171–185 9011393 10.1007/s004410050743

[20] Clevers H (2016) Modeling development and disease with organoids. Cell 165:1586–1597 27315476 10.1016/j.cell.2016.05.082

[21] Corsini NS, Knoblich JA (2022) Human organoids: new strategies and methods for analyzing human development and disease. Cell 185:2756–2769 35868278 10.1016/j.cell.2022.06.051

[22] Simintiras CA, Dhakal P, Ranjit C, Fitzgerald HC, Balboula AZ, Spencer TE (2021) Capture and metabolomic analysis of the human endometrial epithelial organoid secretome. Proc Natl Acad Sci U S A 118:e2026804118 33876774 10.1073/pnas.2026804118 PMC8053979

[23] Boretto M, Cox B, Noben M, Hendriks N, Fassbender A, Roose H, Amant F, Timmerman D, Tomassetti C, Vanhie A, Meuleman C, Ferrante M, Vankelecom H (2017) Development of organoids from mouse and human endometrium showing endometrial epithelium physiology and long-term expandability. Development 144:1775–1786 28442471 10.1242/dev.148478

[24] Saadeldin IM, Han A, Bang S, Kang H, Kim H, Abady MM, Jeong J-S, Kwon H-J, Lee S, Cho J (2024) Generation of porcine endometrial organoids and their use as a model for enhancing embryonic attachment and elongation. Reproduction 167:e230429 38112579 10.1530/REP-23-0429

[25] Turco MY, Gardner L, Hughes J, Cindrova-Davies T, Gomez MJ, Farrell L, Hollinshead M, Marsh SGE, Brosens JJ, Critchley HO, Simons BD, Hemberger M, Koo BK, Moffett A, Burton GJ (2017) Long-term, hormone-responsive organoid cultures of human endometrium in a chemically defined medium. Nat Cell Biol 19:568–577 28394884 10.1038/ncb3516 PMC5410172

[26] Fitzgerald HC, Dhakal P, Behura SK, Schust DJ, Spencer TE (2019) Self-renewing endometrial epithelial organoids of the human uterus. Proc Natl Acad Sci U S A 116:23132–23142 31666317 10.1073/pnas.1915389116 PMC6859318

[27] Spencer TE, Kelleher AM, Bartol FF (2019) Development and function of uterine glands in domestic animals. Annu Rev Anim Biosci 7:125–147 30183326 10.1146/annurev-animal-020518-115321

[28] Wang W, Vilella F, Alama P, Moreno I, Mignardi M, Isakova A, Pan W, Simon C, Quake SR (2020) Single-cell transcriptomic atlas of the human endometrium during the menstrual cycle. Nat Med 26:1644–1653 32929266 10.1038/s41591-020-1040-z

[29] Seishima R, Leung C, Yada S, Murad KBA, Tan LT, Hajamohideen A, Tan SH, Itoh H, Murakami K, Ishida Y, Nakamizo S, Yoshikawa Y, Wong E, Barker N (2019) Neonatal Wnt-dependent Lgr5 positive stem cells are essential for uterine gland development. Nat Commun 10:5378 31772170 10.1038/s41467-019-13363-3 PMC6879518

[30] Xu QX, Zhang WQ, Lu L, Wang KZ, Su RW (2023) Distinguish characters of luminal and glandular epithelium from mouse uterus using a novel enzyme-based separation method. Reprod Sci 30:1867–1877 36581776 10.1007/s43032-022-01154-z

[31] Haider S, Gamperl M, Burkard TR, Kunihs V, Kaindl U, Junttila S, Fiala C, Schmidt K, Mendjan S, Knofler M, Latos PA (2019) Estrogen signaling drives ciliogenesis in human endometrial organoids. Endocrinology 160:2282–2297 31290979 10.1210/en.2019-00314

[32] Chen Y, Antoniou E, Liu Z, Hearne LB, Roberts RM (2007) A microarray analysis for genes regulated by interferon-tau in ovine luminal epithelial cells. Reproduction 134:123–135 17641094 10.1530/REP-07-0387

[33] An SY, Gao XX, Wang ZB, Liang YX, Wang ST, Xiao SH, Xia JT, You PH, Wang F, Zhang GM (2020) Estradiol-17beta regulates proliferation and apoptosis of sheep endometrial epithelial cells by regulating the relative abundance of YAP1. Anim Reprod Sci 215:106328 32216937 10.1016/j.anireprosci.2020.106328

[34] Gilfeather CL, Lemley CO (2016) Effects of interferon-tau and steroids on cytochrome P450 activity in bovine endometrial epithelial cells. Reprod Domest Anim 51:415–420 27103466 10.1111/rda.12695

[35] Gao X, Yao X, Li X, Liang Y, Liu Z, Wang Z, Li K, Li Y, Zhang G, Wang F (2021) Roles of WNT6 in sheep endometrial epithelial cell cycle progression and uterine glands organogenesis. Vet Sci 8:316 34941843 10.3390/vetsci8120316 PMC8708052

[36] Landim L Jr, Miglino MA, Pfarrer C, Ambrósio CE, Garcia J (2007) Culture of mature trophoblastic giant cells from bovine placentomes. Anim Reprod Sci 98:357–364 16716544 10.1016/j.anireprosci.2006.04.053

[37] Shibata S, Endo S, Nagai LAE, E HK, Oike A, Kobayashi N, Kitamura A, Hori T, Nashimoto Y, Nakato R, Hamada H, Kaji H, Kikutake C, Suyama M, Saito M, Yaegashi N, Okae H, Arima T (2024) Modeling embryo-endometrial interface recapitulating human embryo implantation. Sci Adv 10:eadi4819 38394208 10.1126/sciadv.adi4819 PMC10889356

[38] Tian J, Yang J, Chen T, Yin Y, Li N, Li Y, Luo X, Dong E, Tan H, Ma Y, Li T (2023) Generation of human endometrial assembloids with a luminal epithelium using air-liquid interface culture methods. Adv Sci (Weinh) 10:e2301868 37635169 10.1002/advs.202301868 PMC10602567

[39] Spencer TE, Hayashi K, Hu J, Carpenter KD (2005) Comparative developmental biology of the mammalian uterus. Curr Top Dev Biol 68:85–122 16124997 10.1016/S0070-2153(05)68004-0

[40] Spencer TE, Sandra O, Wolf E (2008) Genes involved in conceptus-endometrial interactions in ruminants: insights from reductionism and thoughts on holistic approaches. Reproduction 135:165–179 18239047 10.1530/REP-07-0327

[41] Wang HQ, Liu Y, Li D, Liu JY, Jiang Y, He Y, Zhou JD, Wang ZL, Tang XY, Zhang Y, Zhen X, Cao ZW, Sheng XQ, Yang CF, Yue QL, Ding LJ, Hu YL, Hu ZB, Li CJ, Yan GJ, Sun HX (2023) Maternal and embryonic signals cause functional differentiation of luminal epithelial cells and receptivity establishment. Dev Cell 58:2376–2392 37643613 10.1016/j.devcel.2023.08.004

[42] Bazer FW, Spencer TE, Johnson GA, Burghardt RC, Wu G (2009) Comparative aspects of implantation. Reproduction 138:195–209 19502456 10.1530/REP-09-0158

[43] Li S, Ren Q (2020) Effects of arsenic on wnt/beta-catenin signaling pathway: a systematic review and meta-analysis. Chem Res Toxicol 33:1458–1467 32307979 10.1021/acs.chemrestox.0c00019

[44] Drakhlis L, Devadas SB, Zweigerdt R (2021) Generation of heart-forming organoids from human pluripotent stem cells. Nat Protoc 16:5652–5672 34759383 10.1038/s41596-021-00629-8

[45] Xu Z, Xu X, Yang B, Mi Y, Wang J (2023) 3D sheep rumen epithelial structures driven from single cells in vitro. Vet Res 54:104 37946298 10.1186/s13567-023-01234-1 PMC10636852

[46] Lim W, Bae H, Bazer FW, Song G (2019) Ephrin A1 promotes proliferation of bovine endometrial cells with abundant expression of proliferating cell nuclear antigen and cyclin D1 changing the cell population at each stage of the cell cycle. J Cell Physiol 234:4864–4873 30238980 10.1002/jcp.27275

[47] Adu-Gyamfi EA, Czika A, Liu TH, Gorleku PN, Fondjo LA, Djankpa FT, Ding YB, Wang YX (2021) Ephrin and Eph receptor signaling in female reproductive physiology and pathology. Biol Reprod 104:71–82 32940657 10.1093/biolre/ioaa171

[48] Gu ZY, Jia SZ, Liu S, Leng JH (2020) Endometrial organoids: a new model for the research of endometrial-related diseases. Biol Reprod 103:918–926 32697306 10.1093/biolre/ioaa124 PMC7609820

[49] Achache H, Revel A (2006) Endometrial receptivity markers, the journey to successful embryo implantation. Hum Reprod Update 12:731–746 16982667 10.1093/humupd/dml004

[50] Giudice LC, Liu B, Irwin JC (2025) Endometriosis and adenomyosis unveiled through single-cell glasses. Am J Obstet Gynecol 232:S105–S123 40253075 10.1016/j.ajog.2024.08.043 PMC12282335

[51] Sternberg AK, Izmaylova L, Buck VU, Classen-Linke I, Leube RE (2024) An assessment of the mechanophysical and hormonal impact on human endometrial epithelium mechanics and receptivity. Int J Mol Sci 25:1429 38612536 10.3390/ijms25073726 PMC11011295

[52] Kakni P, Lopez-Iglesias C, Truckenmuller R, Habibovic P, Giselbrecht S (2023) PSC-derived intestinal organoids with apical-out orientation as a tool to study nutrient uptake, drug absorption and metabolism. Front Mol Biosci 10:1102209 36743212 10.3389/fmolb.2023.1102209 PMC9889654

[53] Co JY, Margalef-Catala M, Li X, Mah AT, Kuo CJ, Monack DM, Amieva MR (2019) Controlling epithelial polarity: a human enteroid model for host-pathogen interactions. Cell Rep 26:2509–2520 30811997 10.1016/j.celrep.2019.01.108 PMC6391775

[54] Ahmad V, Yeddula SGR, Telugu B, Spencer TE, Kelleher AM (2024) Development of polarity-reversed endometrial epithelial organoids. Reproduction 167:e230478 38215284 10.1530/REP-23-0478 PMC10959009

[55] Yang T, Zhao J, Liu F, Li Y (2022) Lipid metabolism and endometrial receptivity. Hum Reprod Update 28:858–889 35639910 10.1093/humupd/dmac026

[56] Motta IG, da Silva AG, Feltrin IR, Souza SV, Degan Mattos AC, Morelli KG, Castro T, Nishimura TK, Ginther OJ, Pugliesi G (2025) Effects of estradiol on PGF(2alpha) synthesis and corpus luteum function during early pregnancy in beef heifers. Theriogenology 237:49–60 39970550 10.1016/j.theriogenology.2025.02.014

[57] Dunlap KA, Erikson DW, Burghardt RC, White FJ, Reed KM, Farmer JL, Spencer TE, Magness RR, Bazer FW, Bayless KJ, Johnson GA (2008) Progesterone and placentation increase secreted phosphoprotein one (SPP1 or osteopontin) in uterine glands and stroma for histotrophic and hematotrophic support of ovine pregnancy. Biol Reprod 79:983–990 18667748 10.1095/biolreprod.108.071068 PMC6287637

[58] Bazer FW, Kim J, Song G, Ka H, Tekwe CD, Wu G (2012) Select nutrients, progesterone, and interferon tau affect conceptus metabolism and development. Ann N Y Acad Sci 1271:88–96 23050969 10.1111/j.1749-6632.2012.06741.x PMC3485747

[59] White FJ, Burghardt RC, Hu J, Joyce MM, Spencer TE, Johnson GA (2006) Secreted phosphoprotein 1 (osteopontin) is expressed by stromal macrophages in cyclic and pregnant endometrium of mice, but is induced by estrogen in luminal epithelium during conceptus attachment for implantation. Reproduction 132:919–929 17127752 10.1530/REP-06-0068

[60] Brooks K, Burns GW, Moraes JG, Spencer TE (2016) Analysis of the uterine epithelial and conceptus transcriptome and luminal fluid proteome during the peri-implantation period of pregnancy in sheep. Biol Reprod 95:88 27535962 10.1095/biolreprod.116.141945

[61] Baranda-Avila N, Mendoza-Rodriguez CA, Morimoto S, Langley E, Cerbon M (2009) Molecular mechanism of cell proliferation in rodent uterus during the estrous cycle. J Steroid Biochem Mol Biol 113:259–268 19429431 10.1016/j.jsbmb.2009.01.008

[62] Chaney HL, Grose LF, Charpigny G, Behura SK, Sheldon IM, Cronin JG, Lonergan P, Spencer TE, Mathew DJ (2021) Conceptus-induced, interferon tau-dependent gene expression in bovine endometrial epithelial and stromal cells. Biol Reprod 104:669–683 33330929 10.1093/biolre/ioaa226

[63] Seo H, Frank JW, Burghardt RC, Bazer FW, Johnson GA (2020) Integrins and OPN localize to adhesion complexes during placentation in sheep. Reproduction 160:521–532 32668403 10.1530/REP-20-0273

[64] Ramos-Ibeas P, Gonzalez-Brusi L, Used MT, Cocero MJ, Marigorta P, Alberio R, Bermejo-Alvarez P (2022) In vitro culture of ovine embryos up to early gastrulating stages. Development 149:dev199743 35319748 10.1242/dev.199743 PMC8977095

[65] Nakamura K, Kusama K, Bai R, Sakurai T, Isuzugawa K, Godkin JD, Suda Y, Imakawa K (2016) Induction of IFNT-stimulated genes by conceptus-derived exosomes during the attachment period. PLoS One 11:e0158278 27351483 10.1371/journal.pone.0158278 PMC4924817

[66] Chen X, Tang L, Pan Y, Xie Y, Jin H, Xiang X, Wang Z (2025) oar-miR-29a promotes the establishment of endometrial receptivity by targeting CDC42 in sheep. Theriogenology 237:22–32 39956034 10.1016/j.theriogenology.2025.02.010

[67] Wooding FB (1984) Role of binucleate cells in fetomaternal cell fusion at implantation in the sheep. Am J Anat 170:233–250 6465051 10.1002/aja.1001700208

[68] Hansen PJ (2007) Regulation of immune cells in the uterus during pregnancy in ruminants. J Anim Sci 85:E30-31 17322123 10.2527/jas.2006-487

[69] Burghardt RC, Burghardt JR, Taylor JD 2nd, Reeder AT, Nguen BT, Spencer TE, Bayless KJ, Johnson GA (2009) Enhanced focal adhesion assembly reflects increased mechanosensation and mechanotransduction at maternal-conceptus interface and uterine wall during ovine pregnancy. Reproduction 137:567–582 19060096 10.1530/REP-08-0304

[70] Saorin G, Caligiuri I, Rizzolio F (2023) Microfluidic organoids-on-a-chip: the future of human models. Semin Cell Dev Biol 144:41–54 36241560 10.1016/j.semcdb.2022.10.001

[71] Gnecco JS, Brown A, Buttrey K, Ives C, Goods BA, Baugh L, Hernandez-Gordillo V, Loring M, Isaacson KB, Griffith LG (2023) Organoid co-culture model of the human endometrium in a fully synthetic extracellular matrix enables the study of epithelial-stromal crosstalk. Med 4:554-579.e9 37572651 10.1016/j.medj.2023.07.004 PMC10878405

