# Supplementary figures and images for: Development and characterization of functional sheep endometrial luminal epithelial organoids
Source: Vet Res. 2026 Jun 9;57:102. doi: 10.1186/s13567-026-01764-4 (PMC13248463; doi:10.1186/s13567-026-01764-4)

Figure S1

**
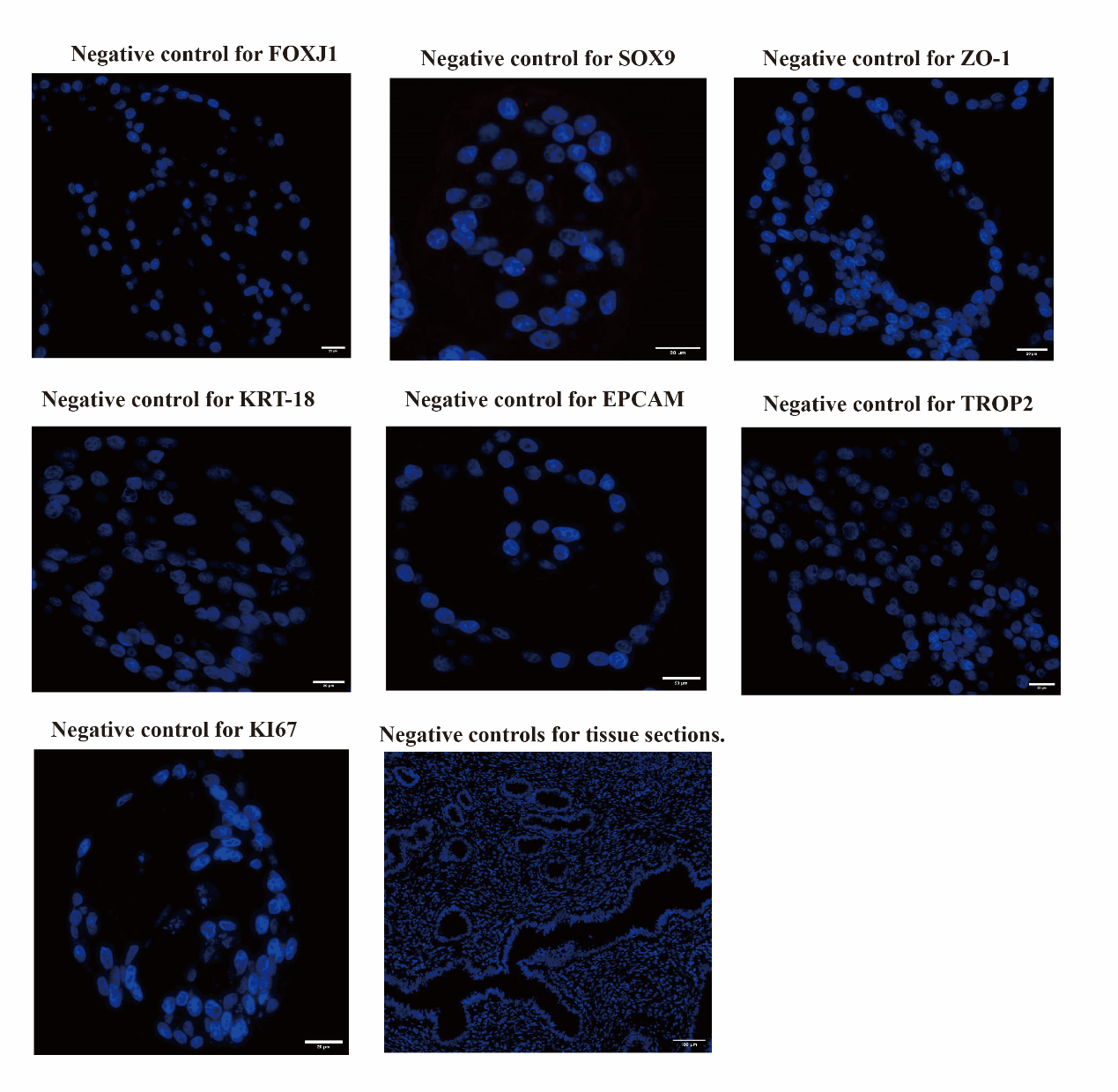
**

**Figure S1.** **The negative controls of various antibodies used in the article**

Supplement: Supplementary file 4 — Additional file 4 Negative controls for immunofluorescence staining in organoids. [file 13567_2026_1764_MOESM4_ESM.docx]

Figure S5


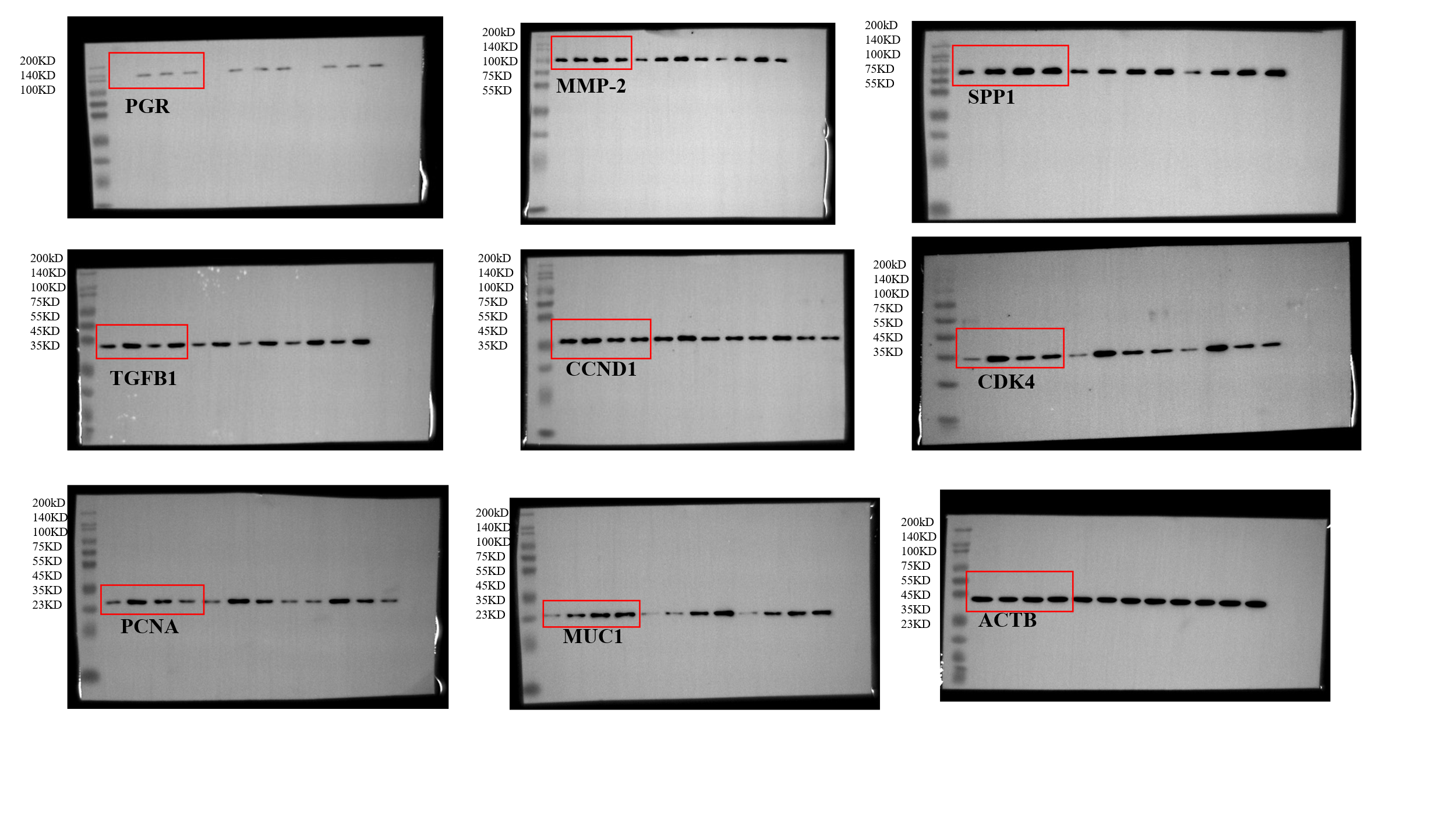


**Figure S5 Original untrimmed images of all results from Western blot analysis**

Supplement: Supplementary file 8 — Additional file 8 Original uncropped images of all Western blot results. [file 13567_2026_1764_MOESM8_ESM.docx]
