# Supplementary material for: Development and characterization of functional sheep endometrial luminal epithelial organoids
Source: Vet Res. 2026 Jun 9;57:102. doi: 10.1186/s13567-026-01764-4 (PMC13248463; doi:10.1186/s13567-026-01764-4)
Supplement: Supplementary file 5 — Additional file 5 Quality assessment of total RNA by microcapillary electrophoresis in organoids under different treatments. RIN > 9.0. [file 13567_2026_1764_MOESM5_ESM.docx]

Figure S2


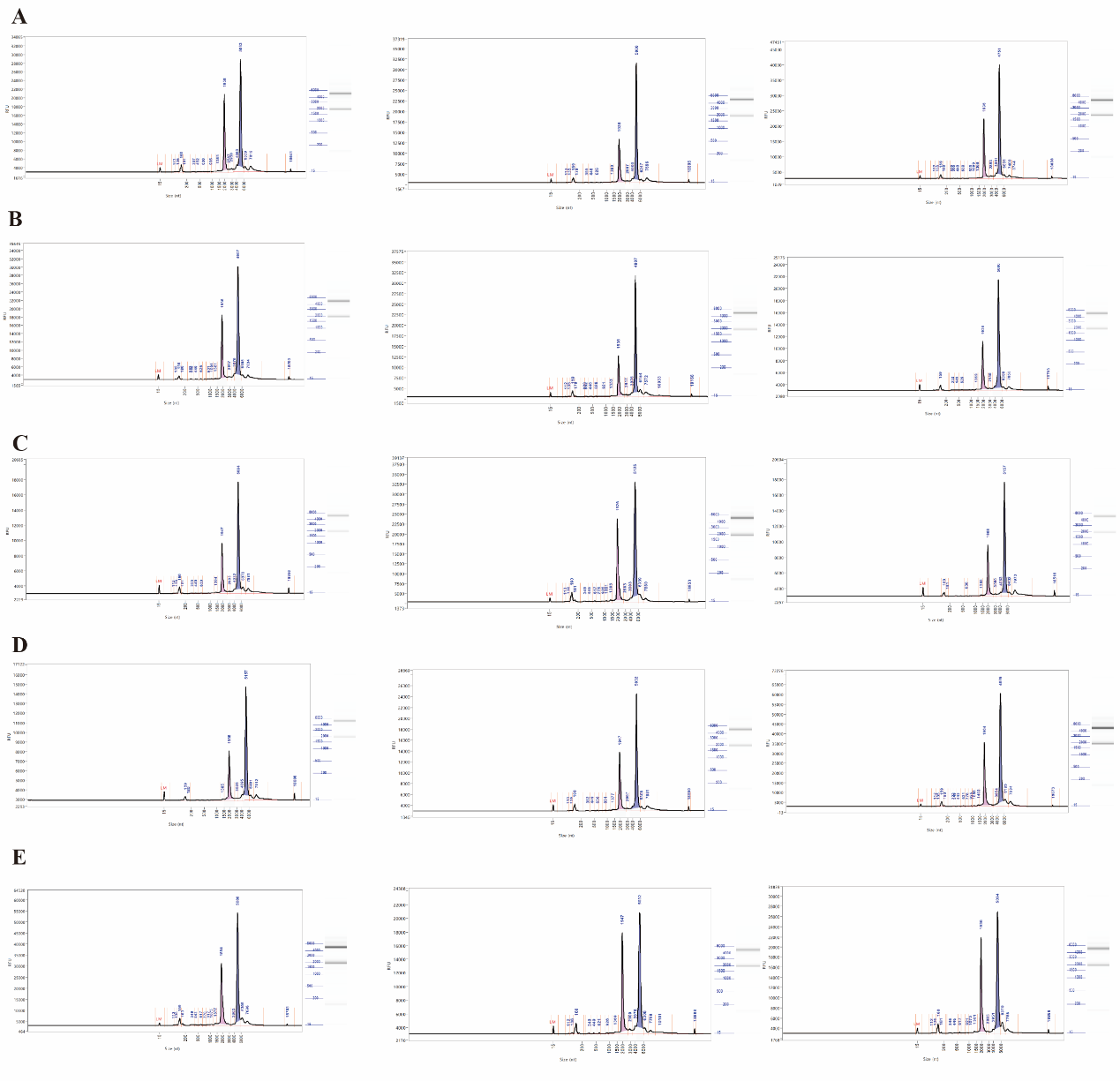


**Figure S2. Quality assessment of total RNA by microcapillary electrophoresis.
A. Representative electropherograms of three biological replicates of control organoids.
B. Representative electropherograms of three biological replicates of organoids cultured in complete medium lacking EPHA1.
C. Representative electropherograms of three biological replicates of organoids treated with estrogen (E2).
D. Representative electropherograms of three biological replicates of organoids treated with E2 + medroxyprogesterone acetate (MPA).
E. Representative electropherograms of three biological replicates of organoids treated with E2 + MPA + interferon tau (IFN-tau).**

**All electropherograms display sharp, distinct 18S and 28S ribosomal RNA peaks with stable baselines and no detectable degradation products, which is fully consistent with RIN > 9.0.**
