# Supplementary material for: Development and characterization of functional sheep endometrial luminal epithelial organoids
Source: Vet Res. 2026 Jun 9;57:102. doi: 10.1186/s13567-026-01764-4 (PMC13248463; doi:10.1186/s13567-026-01764-4)
Supplement: Supplementary file 6 — Additional file 6 Immunohistochemical identification of ovine trophoblast cells (positive for CK-7, GATA3, and FABP3). [file 13567_2026_1764_MOESM6_ESM.docx]

Figure S3


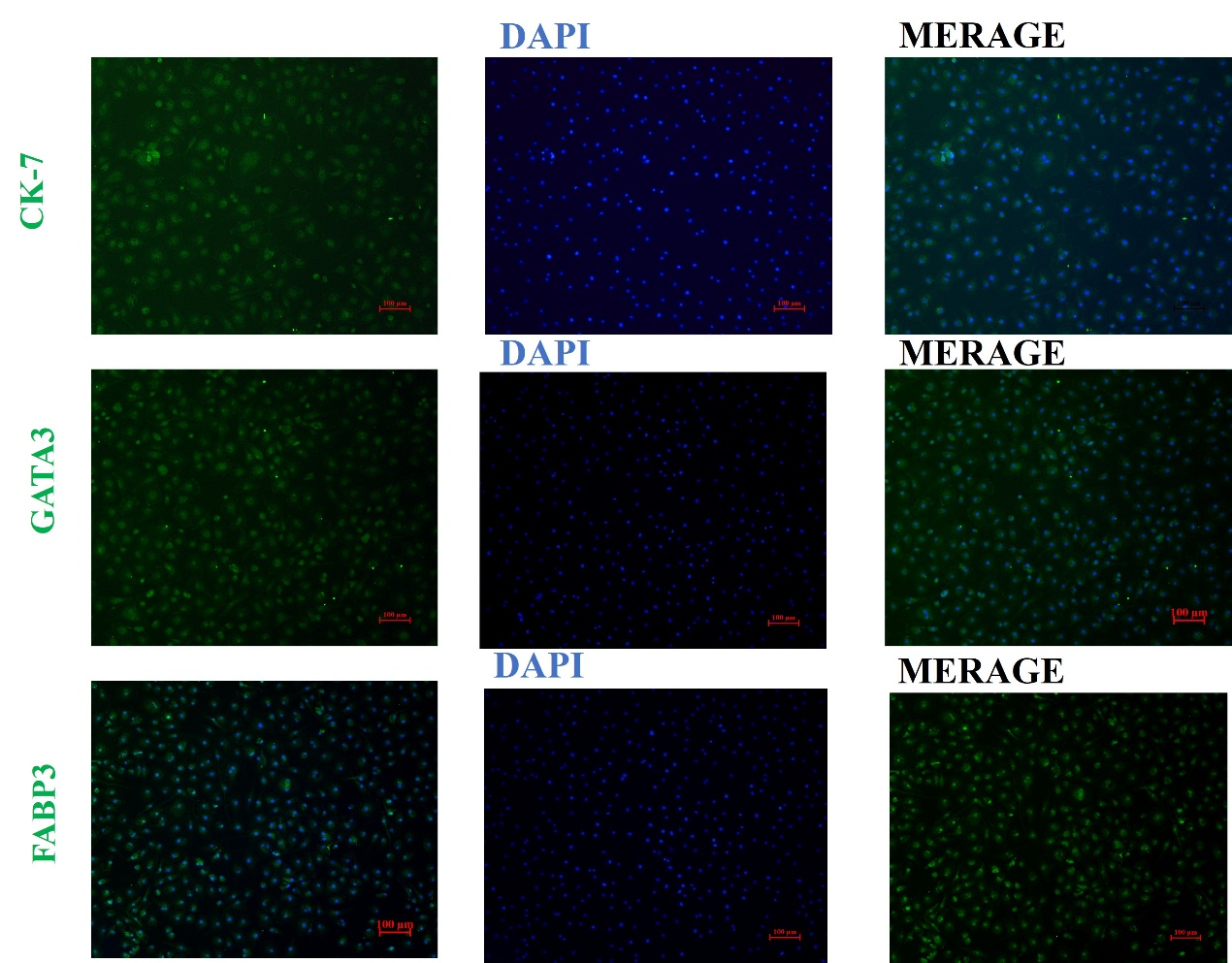


**Figure S3. Identification of ovine trophoblast cells by immunohistochemical markers.**
**Positive staining for CK-7, GATA3, and FABP3 confirms trophoblast cell identity.**
