# Supplementary material for: Development and characterization of functional sheep endometrial luminal epithelial organoids
Source: Vet Res. 2026 Jun 9;57:102. doi: 10.1186/s13567-026-01764-4 (PMC13248463; doi:10.1186/s13567-026-01764-4)
Supplement: Supplementary file 7 — Additional file 7 Immunofluorescence staining of FOXA2 and corresponding FPKM values from transcriptome sequencing in ovine organoids. [file 13567_2026_1764_MOESM7_ESM.docx]

Figure S4


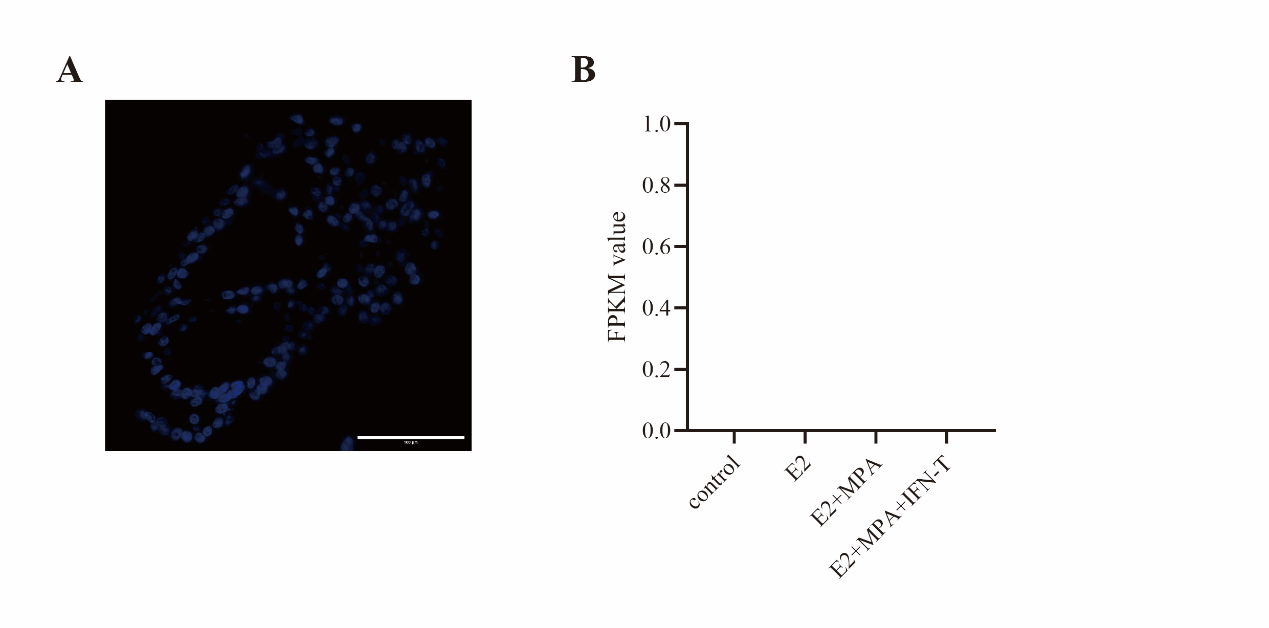


**Figure S4. The immunofluorescence staining of FOXA2 and the FPKM values obtained from transcriptome sequencing**

1. **The immunofluorescence staining of FOXA2**
2. **The FPKM values of FOXA2 in the transcriptome sequencing data across the four experimental groups: Control, E2, E2+MPA, and E2+MPA+IFN-τ.**
