# Supplementary material for: Development and characterization of functional sheep endometrial luminal epithelial organoids
Source: Vet Res. 2026 Jun 9;57:102. doi: 10.1186/s13567-026-01764-4 (PMC13248463; doi:10.1186/s13567-026-01764-4)
Supplement: Supplementary file 9 — Additional file 9 Ct values from no-reverse transcription controls (−RT) and experimental cDNA groups for all target genes. [file 13567_2026_1764_MOESM9_ESM.docx]

Table S1

**TABLE1. Ct values from the no-reverse transcription control (-RT) and experimental cDNA groups for target genes**

| **Gene name** | **+RT sample (Ct value)** | **-RT control (Ct value)** | **ΔCt (control - experimental)** | **Conclusion** |
| --- | --- | --- | --- | --- |
| **SPP1** | 22.5 ± 0.3 | Undetected | N/A | No gDNA contamination |
| **PGR** | 28.7±0.42 | Undetected | N/A | No gDNA contamination |
| **TGFB1** | 20.9±0.29 | Undetected | N/A | No gDNA contamination |
| **MMP2** | 26.2±0.19 | 35.1 ± 0.22 | ΔCt =8.9 ± 0.29 | No gDNA contamination |
| **PCNA** | 19.3±0.25 | Undetected | N/A | No gDNA contamination |
| **CCND1** | 23.7±0.33 | 36.2±0.31 | ΔCt =12.5 ± 0.453 | No gDNA contamination |
| **CDK4** | 29.5±0.45 | Undetected | N/A | No gDNA contamination |
| **ACTB** | 17.3±0.29 | Undetected | N/A | No gDNA contamination |
